# Soil-plant microbiomes shaped by soil type and wastewater quality as shown by a combination of cultivation-dependent and independent techniques

**DOI:** 10.1093/femsec/fiag087

**Published:** 2026-08-12

**Authors:** Sara Gallego, Leila Soufi, Ioannis D Kampouris, Rehana Abdulvakkeil, Dipen Pulami, Kathia Lüneberg, Benjamin J Heyde, Doreen Babin, Christina Siebe, Jan Siemens, Stefanie P Glaeser, Kornelia Smalla, Elisabeth Grohmann

**Affiliations:** Julius Kühn Institute (JKI) - Federal Research Centre for Cultivated Plants, Institute for Epidemiology and Pathogen Diagnostics, 38104 Braunschweig, Germany; Berlin University of Applied Sciences, Faculty of Life Sciences and Technology, Department of Microbiology, 13353 Berlin, Germany; Julius Kühn Institute (JKI) - Federal Research Centre for Cultivated Plants, Institute for Epidemiology and Pathogen Diagnostics, 38104 Braunschweig, Germany; Justus Liebig University Giessen, iFZ Research Center for BioSystems, Land Use and Nutrition, Institute of Soil Science and Soil Conservation, 35392 Giessen, Germany; Justus Liebig University Giessen, iFZ Research Center for BioSystems, Land Use and Nutrition, Institute of Soil Science and Soil Conservation, 35392 Giessen, Germany; Instituto de Geología, Universidad Nacional Autónoma de México, Ciudad Universitaria, 04510 México City, México; Justus Liebig University Giessen, iFZ Research Center for BioSystems, Land Use and Nutrition, Institute of Soil Science and Soil Conservation, 35392 Giessen, Germany; Ruhr University Bochum, Institute of Geography, Unit Soil Sciences and Soil Resources, 44801 Bochum, Germany; Julius Kühn Institute (JKI) - Federal Research Centre for Cultivated Plants, Institute for Epidemiology and Pathogen Diagnostics, 38104 Braunschweig, Germany; Instituto de Geología, Universidad Nacional Autónoma de México, Ciudad Universitaria, 04510 México City, México; Justus Liebig University Giessen, iFZ Research Center for BioSystems, Land Use and Nutrition, Institute of Soil Science and Soil Conservation, 35392 Giessen, Germany; Justus Liebig University Giessen, iFZ Research Center for BioSystems, Land Use and Nutrition, Institute of Soil Science and Soil Conservation, 35392 Giessen, Germany; Julius Kühn Institute (JKI) - Federal Research Centre for Cultivated Plants, Institute for Epidemiology and Pathogen Diagnostics, 38104 Braunschweig, Germany; Berlin University of Applied Sciences, Faculty of Life Sciences and Technology, Department of Microbiology, 13353 Berlin, Germany

**Keywords:** antibiotic resistance genes, mobile genetic elements, rhizosphere, phyllosphere, Mezquital Valley, third-generation cephalosporin-resistant enterobacteria

## Abstract

The effects of wastewater irrigation on antibiotic resistance genes (ARGs), mobile genetic elements (MGEs), and bacterial communities in plant-associated microbiomes and soils are still poorly understood. We conducted a column experiment using Leptosol and Vertisol monoliths, planted with cilantro (*Coriandrum sativum*) and irrigated for eight weeks with untreated or treated (TWW) wastewater, with or without spiked antibiotics and disinfectants. Total community-DNA was analyzed by qPCR and 16S rRNA gene sequencing. Third-generation cephalosporin-resistant (3GCR) enterobacteria were isolated. Spiked WW irrigation increased ARG and MGE relative abundances in preferential flow path soil. In the rhizosphere, Leptosol exhibited higher ARG and MGE relative abundances than Vertisol. Spiking increased relative abundances of class 1 integrons, sulfonamide, tetracycline resistance genes in soil and rhizosphere, and erythromycin and fluoroquinolone resistance genes in phyllosphere. Soil type primarily shaped bacterial community composition in preferential flow path soil and rhizosphere, followed by spiking, whereas WW type influenced only rhizosphere bacterial community composition. 3GCR strains of the *Citrobacter freundii* complex genotype Ci-1a_Bx-C2 were isolated exclusively from soil and rhizosphere enrichments irrigated with (spiked or unspiked) TWW. We demonstrate the need to combine cultivation-independent and cultivation-based methods to investigate the effects of WW irrigation on soil and plant-associated microbiomes.

## Introduction

The growing adoption of wastewater (WW) reuse for agricultural irrigation has emerged as a strategic response to global water scarcity (Christou et al. 2024). Treated wastewater (TWW) offers a reliable alternative to freshwater, supporting crop production in water-stressed regions (Ofori et al. 2021, Heyde et al. 2025). However, despite advances in WW treatment technologies, residual micropollutants—including antibiotics and disinfectants—along with antibiotic-resistant bacteria (ARB) carrying antibiotic resistance genes (ARGs), may persist in WW even after treatment (Rizzo et al. 2013, Hendriksen et al. 2019, Pärnänen et al. 2019, Pazda et al. 2019, Matesun et al. 2024). Once introduced into agricultural soils, WW-borne micropollutants can alter both physicochemical and microbial soil properties (Siebe and Fischer 1996, Becerra-Castro et al. 2015) and exert a selective pressure on soil and plant-associated microbial communities (Smalla et al. 2023). Additionally, the co-occurrence of these micropollutants with bacteria harbouring ARGs localized on mobile genetic elements (MGEs), such as plasmids and integrons, facilitates their horizontal transfer, contributing to the emergence and dissemination of antimicrobial resistance among environmental bacteria and potential pathogens (Heuer and Smalla 2012, Larsson and Flach 2021, Bengtsson-Palme et al. 2023, Murray et al. 2024). This raises serious concerns about the potential transmission of resistance traits and pathogens into the food chain (Pan and Chu 2017, Blau et al. 2018, Gekenidis et al. 2018, FAO and WHO 2019, Gudda et al. 2020, Hirt 2020).

Studies evaluating the impact of reclaimed WW irrigation on the occurrence and dissemination of ARGs and MGEs in soil-plant systems have reported inconsistent findings. While some studies observed changes in ARG diversity, distribution, and abundance along the soil-plant continuum (Cerqueira et al. 2019a,b, Bhattacharjee et al. 2024, Phan et al. 2024), others reported minor or no effects of irrigation water quality on ARGs and MGEs (Cerqueira et al. 2019c, Marano et al. 2019, Ofori et al. 2025). These contrasting outcomes suggest that the mechanisms governing ARG dissemination in agricultural systems remain poorly understood and may depend on additional factors, including soil properties, crop species, and agricultural management practices.

In addition to increasing antimicrobial resistance, reclaimed WW irrigation can disrupt natural soil-plant microbial communities and introduce human pathogens into plant microbiomes, posing significant risks to human health (Alegbeleye et al. 2018, Bhattacharjee et al. 2024). While several studies have demonstrated that WW irrigation influences the composition and diversity of root- and plant-associated bacterial communities (Cerqueira et al. 2019a, b, Zolti et al. 2019, Bigott et al. 2022), Cerqueira et al. (2019c) observed that TWW had a smaller or negligible effect on soil and plant microbiomes compared with fertilization or organic amendments.

The Mezquital Valley in central Mexico offers a unique opportunity to shed light on the influence of irrigation water quality on ARGs, MGEs, and microbial communities of soil-plant systems. Over the past century, untreated wastewater (UWW) from Mexico City has been used for agricultural irrigation, resulting in a continuous input of pharmaceuticals, heavy metals, and disinfectants, among others, into the soils (Siebe and Fischer 1996, Lucho-Constantino et al. 2005, Siemens et al. 2008, Dalkmann et al. 2012, Heyde et al. 2021). Studies in the area have also reported high abundances of potential pathogens and elevated levels of widespread, clinically relevant ARGs, including tetracycline (*tetW, tetQ*), ciprofloxacin (*qnrB* and *qnrS*), sulfonamide (*sul1, sul2*), and streptomycin (*aadA*) resistance genes in the soils (Dalkmann et al. 2012, Jechalke et al. 2015, Broszat and Grohmann 2017, Lüneberg et al. 2018b). Along with those, class 1 integrons (*intl1*), indicators of anthropogenic pollution (Gillings et al. 2015), and MGEs such as IncP-1 plasmid-backbone genes (*korB*), broad host-range plasmids that facilitate horizontal gene transfer across bacterial species, were also detected (Jechalke et al. 2015). Despite extensive evidence of long-term accumulation of contaminants and resistance determinants in Mezquital Valley soils, it remains unclear to what extent irrigation water quality directly shapes ARG and MGE distribution and alters bacterial community composition across the soil–plant continuum. Little is known about whether these effects differ between soil and plant compartments, depend on soil type, or influence the occurrence of clinically relevant antibiotic-resistant bacteria associated with crops.

Controlled soil column experiments offer a valuable approach to investigate the effects of WW irrigation on soil-plant systems under defined conditions while preserving soil structure. In particular, they enable the study of preferential flow paths in soils, which are of particular concern due to their role in pollutant accumulation and transport (Reichenberger et al. 2002, Kim et al. 2008, Salazar-Ledesma et al. 2018, Zhang et al. 2018), and in the dissemination of antibiotic resistance (Lüneberg et al. 2018a). Therefore, this study aimed to determine how irrigation water quality influences ARG and MGE relative abundance and shapes bacterial community composition along the soil-plant continuum, considering additionally the influence of soil type. We hypothesized that WW-borne pollutants, in particular antibiotics and disinfectants, promote the selection and dissemination of bacteria carrying ARGs and MGEs, with effects varying along the soil-plant continuum in a soil-type-dependent manner. To test this, we conducted a greenhouse experiment using monolithic soil columns of two representative soil types (Leptosols and Vertisols) from the Mezquital Valley planted with cilantro and irrigated for eight weeks with UWW and TWW spiked or not with a mixture of antibiotics and disinfectants. The influence of irrigation water quality (water type and spiking level) on ARGs and MGEs abundance was evaluated in total community (TC)-DNA using real-time qPCR, while the effects on the bacterial community composition in soil–plant systems were assessed by 16S rRNA gene amplicon sequencing. To capture clinically relevant ARBs that are typically below the limit of detection using DNA-based methods, selective enrichment, isolation, and characterization of third-generation cephalosporin-resistant (3GCR) enterobacteria were performed from the same samples. Antibiotics and disinfectants were investigated in the same samples but reported separately (Heyde et al. under revision).

## Materials and methods

### WW collection and analysis

UWW and TWW were collected from the Atotonilco WWTP influent and effluent, respectively (19°57′28.52“N, 99°17′51.02″W), in February 2023 and stored in 5000 l tanks at ambient temperature at the lysimeter station (19°18′42.3“N, 99°10′36.8″W) of the National Autonomous University of Mexico (UNAM) for the duration of the experiment (8 weeks). The WWTP operates using conventional activated sludge biological treatment without dedicated nitrification or denitrification units, to preserve nutrients for agricultural reuse in the Mezquital Valley (CONAGUA 2007, Garduño-Jiménez et al. 2023). WW samples were collected from the tanks monthly (five samples each from UWW and TWW) and analyzed for the following parameters: pH was measured with a potentiometer Hanna HI2002-01 (Hanna Instruments, RI, USA), electrical conductivity (EC) with a conductivity meter Hanna Edge (Hanna Instruments, RI, USA), turbidity with a nephelometer HI98703 (Hanna Instruments, RI, USA) adapted for US EPA 180.1 method (O’Dell 1996), calibrated from 0 to 750 nephelometric turbidity units (NTU), chemical oxygen demand (COD) (DIN—German Institute for Standardization 1980), total organic carbon (TOC) (DIN—German Institute for Standardization 2019), total dissolved nitrogen (N_tot_), nitrate, ammonium, dissolved organic nitrogen (N_org_), and total phosphorus. N_tot_, nitrate, and ammonium were measured using a Seal AutoAnalyzer 500. N_tot_ (Method AD-077–20) was determined by alkaline UV oxidation with potassium peroxydisulfate, followed by reduction with hydrazine/Cu and colourimetric detection at 540 nm after reaction with sulfonamide and N-(1-naphthyl) ethylenediamine. Nitrate was measured similarly without prior oxidation (Method AD-068–19 Rev. 1). Ammonium was determined at 660 nm after reaction with salicylate and chlorine, using sodium nitroprusside as a catalyst (Method AD-048–19). N_org_ was calculated as the difference between N_tot_ and the sum of nitrate-N (NO₃-N) and ammonium-N (NH₄-N) concentrations. Total phosphorus and heavy metal concentrations were measured by ICP-OES after HNO₃ digestion of WW samples (Table 1). Concentrations of selected antibiotics, anhydroerythromycin (a degradation product of erythromycin), azithromycin, ciprofloxacin, clindamycin, erythromycin, sulfamethoxazole, and trimethoprim (Table 1) were assessed as described in Soufi et al. (2025).

**Table 1:** Physicochemical characteristics of UWW and TWW used to irrigate the soil columns and concentrations of spiked antibiotics and disinfectants used in this study.

Parameters	UWW	TWW	Spiked concentrations
pH	7.90	7.88	
EC (µS/cm)	1352.25	1439.75	
Turbidity (NTU)	7.79	2.59	
COD [mg/l]	83	46	
TOC [mg/l]	20.1	11.1	
N_tot_ [mg N/l]	19.5	23.03	
Nitrate [mg N/l]	0.04	4.1	
Ammonium [mg N/l]	16.9	18.8	
N_org_ [mg N/l]	2.56	0.13	
Total phosphorus [mg P/l]	14.43	2.73	
Anhydroerythromycin [ng/l]*	19	118	
Azithromycin [ng/l]	0.5	0.7	695 000
Ciprofloxacin [ng/l]	100.5	28.6	1695 000
Clindamycin [ng/l]	18	160	60 000
Erythromycin [ng/l]	< LOD	< LOD	185 000
Sulfamethoxazole [ng/l]	2966	502	1 995 000
Trimethoprim [ng/l]	305	752	650 000
ATMACs [ng/l]	–**	–	84 546
BACs [ng/l]	–	–	261 650
DADMACs [ng/l]	–	–	156 452

* Anhydroerythromycin = degradation product of erythromycin.

** :—not measured.

LOD: limit of detection.

EC: electrical conductivity; COD: chemical oxygen demand; TOC: total organic carbon; N_tot_: total dissolved nitrogen; N_org_: dissolved organic nitrogen; ATMACs: alkyltrimethyl ammonium compounds; BACs: benzylalkyldimethyl ammonium compounds; DADMACs: dialkyldimethyl ammonium compounds.

### Experimental design of the soil column experiment

The soil column experiment was conducted in a greenhouse at UNAM in Mexico City. Monolithic (undisturbed) soil cores from the upper 20 cm of the soil were collected from two representative soil types in the Mezquital Valley—Leptosol (Tlaxcoapan) and Vertisol (Ulapa de Melchor Ocampo)—irrigated with UWW for over 90 years (Fig. S1) (Siebe 1994, Bonvehi Rosich and Denizen 2025). Physicochemical properties of air-dried soil samples were characterized following the methodology described in Soufi et al. (2025) (Table 2).

**Table 2:** Physicochemical characteristics of the soils used in the soil column experiment.

Parameters	Leptosol	Vertisol
Sand [%]	62	13
Silt [%]	25	34
clay [%]	13 (sandy loam)	53 (clay)
C_tot_ [%]	2.33	2.70
C_org_ [%]	2.16	2.61
Carbonate C [%]	0.17	0.09
N_tot_ [%]	0.21	0.26
S_tot_ [%]	0.072	0.067
pH	7.01	7.22
EC (µS cm^−1^)	317	357

C_tot_: total carbon; C_org_: organic carbon; carbonate C: inorganic carbon; N_tot_: total nitrogen; S_tot_: total sulfur; EC: electrical conductivity determined in 1 : 10 (w: vol) water extract.

A total of 16 soil monoliths were collected per soil type (32 columns in total) by manually inserting plastic tubes (15 cm inner diameter, 22 cm depth). The monoliths were extracted by cutting the base with a knife and transported to the greenhouse. To minimize pore smearing and improve drainage, the bottom 2 cm of each monolith were replaced with washed quartz sand (mean grain size = 0.2 mm). Cilantro (Hortaflor variety) seeds were grown in sterile conditions. After germination, twenty sprouts were planted in each column. Cilantro was chosen as the model plant as it is grown in the Mezquital Valley and eaten raw. Cilantro is sensitive to WW-derived contaminants and can accumulate heavy metals and pollutants from irrigation water, and may act as a reservoir for transferable ARGs, making it a suitable model for studying WW impacts on soil–plant systems (Assadian et al. 1998, Blau et al. 2018, Atamaleki et al. 2021). Soil columns were kept under controlled conditions (20.5 ± 2.5°C, 40%–80% humidity, light intensity from 13 to 40 W/m² for 12 h a day) for eight weeks to allow the plants to grow.

The soil columns were irrigated with either unspiked UWW (I), spiked UWW (II), unspiked TWW (III), or spiked TWW (IV), with four replicates per treatment and soil type. Spiking was performed by adding a mixture of antibiotics (60 µg/l—1995 µg/l) (azithromycin, ciprofloxacin, clindamycin, erythromycin, sulfamethoxazole, trimethoprim (HPC Standards GmbH), and disinfectants (4 µg/l—159 µg/l) (alkyltrimethyl ammonium compounds (ATMACs), benzylalkyldimethyl ammonium compounds (BACs), and dialkyldimethyl ammonium compounds (DADMACs), TCI Deutschland GmbH) to the UWW and TWW before each irrigation event. The concentrations of spiked antibiotics and disinfectants were 500-fold higher than those previously detected in WWTP influent samples (Soufi et al. 2025). Spiking was performed to simulate the cumulative effects of continuous and long-term irrigation events.

The columns were irrigated twice a week to ensure adequate soil moisture and to prevent wilting. WW was manually applied directly to the soil near the plant base, avoiding contact with the leaves. During weeks 1–5, Leptosol columns received 2 × 360 ml and Vertisol 2 × 440 ml of WW per week. From weeks 6–8, irrigation increased to 2 × 460 ml for Leptosol and 2 × 640 ml for Vertisol. Due to their higher clay content and water retention capacity, Vertisol columns received larger irrigation volumes than Leptosols to achieve comparable soil water exchange rates in both soil types and to reflect local irrigation practices in the Mezquital Valley (Table S1). Brilliant blue (3 g/l Erioglaucine; Sigma–Aldrich) was added during the final two irrigation events to stain the soil in contact with percolating water in all treatments (I–IV) to visualize the preferential flow paths (Lüneberg et al. 2018a).

### Soil, rhizosphere, and phyllosphere sampling and processing

One week after the final irrigation event, cilantro plants and soil samples were collected. The aboveground parts of the plants were cut with scissors, and root systems were extracted by cutting the plastic columns with a rotary microtool (Dremel, Racine, USA) and splitting the soil monoliths longitudinally with a sterile knife (Fig. S2). Blue-stained soil exposed to irrigation water percolating through preferential flow paths (stained soil) and unstained soil (bulk soil not or less exposed to irrigation water) were sampled separately using a sterile spatula as a composite sample across the whole column length. Identification and separation of stained and unstained regions followed an established procedure described by Lüneberg et al. (2018a). They were performed by colour thresholding using the image analysis software Fiji, as described in Heyde et al. (under revision). For cultivation analysis, portions of the composite samples were mixed with buffered peptone water (BPW; Sigma-Aldrich) containing 20% (v/v) glycerol and stored at −20°C. Remaining portions were stored at 4°C for chemical analyses or −20°C for DNA-based analyses.

Loosely bound soil was shaken off the roots. Cilantro plants (separated into shoots and roots) were weighed. Soil remaining attached to roots after shaking was defined as the rhizosphere. Bacterial cells from the rhizosphere and phyllosphere (leaves) were detached following Blau et al. (2019) with minor modifications. Briefly, two grams of roots with adhering rhizosphere or 5 g of leaves (phyllosphere) per column were combined into a homogeneous sample and mixed at 1 : 10 ratio with sterile phosphate buffer (PPB, 49 mM KH_₂_PO_₄_, 50 mM K_₂_HPO_₄_). Samples were vortex-mixed at maximum speed for 1 min, and supernatants collected. This procedure was repeated twice (in total: 3 × 6 ml for rhizosphere or 3 × 15 ml for phyllosphere). The supernatants from each sample were pooled. An aliquot (1 ml) of each pooled sample was mixed with 1 ml BPW containing 20% (v/v) glycerol. The samples were stored at −20°C for cultivation-dependent analysis. The remaining pooled supernatants were centrifuged at 3100 × *g* for 15 mins at 4°C. The resulting pellets were stored at −20°C for subsequent DNA extraction.

### DNA extraction, detection, and quantification of target genes via real-time qPCR

WW samples (40–50 ml of UWW and 60–100 ml of TWW; five samples each) were filtered through sterile polyether sulfone membrane Millipore Sterivex filter units (0.22 μm pore size; Merck, Darmstadt, Germany). The Sterivex cartridges were opened, the filters were removed, and placed into the lysis tubes of the DNA extraction kit. Filters, stained and unstained soil, rhizosphere, and phyllosphere samples were used for TC-DNA extraction with the FastDNA SPIN Kit for Soil (MP Biomedicals, Heidelberg, Germany) following the manufacturer’s instructions. Extracted TC-DNA was stored at −20°C until further analysis. Selected genes were quantified from TC-DNA extracted from all samples using qPCR (TaqMan qPCR) on a CFX96 real-time PCR detection system (Bio-Rad, Hercules, CA, United States). They included 16S rRNA genes, erythromycin (*ermA* and *ermB*), fluoroquinolone (*qnrA* and *qnrS*), sulfonamide (*sul1* and *sul2*), tetracycline (*tetA* and *tetM)* resistance genes, as well as the class 1 integron integrase gene (*intI1)*. In addition, the presence of the IncP-1 plasmids-specific *korB* gene, gene markers for Inc18 plasmids (*inc18*), *Enterococcus* plasmids (*repA_N*), pSK1 (*rep*_pSK1_), and pI258 (*rep*_pI258_) families of *Staphylococcus* plasmids were quantified. qPCR assays were performed in 25 µl reaction mixtures containing 0.625 U of HotStart Taq polymerase (New England Biolabs, Germany), 0.2–1.2 µM of each primer, 0.2 mM of each dNTP, 0.1 mg/ml bovine serum albumin (BSA), 1x HotStart Taq buffer, 1.5–4 mM MgCl_2_, and 5 µl of DNA template (1 : 5 dilution for ARGs and 1 : 50 dilution for 16S rRNA gene). TaqMan assays included 0.3–0.5 µM TaqMan probe, while EvaGreen assays (*qnrA* and *qnrS*) used 1 × EvaGreen, with melting curve analysis for amplicon specificity. Primer sequences and qPCR conditions are provided in Supplementary Table S2. Each run included a 10-fold dilution standard curve (10¹–10¹⁰ gene copies) with efficiencies of 90%–110% and R² ≥ 0.98. PCR-grade water served as a negative control. Relative gene abundance was calculated as the number of target gene copies per 16S rRNA gene copies.

### 16S rRNA gene amplicon sequencing analysis

TC-DNA from WW, soil, rhizosphere, and phyllosphere was used for amplification and sequencing of 16S rRNA gene fragments. The library was constructed and sequenced by Novogene (Cambridge, UK) on a NovaSeq 6000 PE250 platform using the 16S rRNA gene-targeting primers Uni341F (5′-CCTAYGGGRBGCASCAG-3′) and Uni806R (5′- GGACTACNNGGGTATCTAAT-3′), targeting bacteria and archaea (Sundberg et al. 2013). Primers and adapters were removed using cutadapt (Martin 2011). Paired-end reads were processed in R (R Core Team 2021; v.4.2.1) using the DADA2 pipeline (Callahan et al. 2016). The obtained amplicon sequence variants (ASVs) were taxonomically classified to the lowest possible taxonomic level by using a Naive Bayesian classifier (Wang et al. 2007), trained on the SILVA SSU Reference Taxonomy database (Quast et al. 2013; v138.1). Decontamination was performed based on the regression to DNA concentrations method described in Davis et al. (2018), with the modification of using Spearman rank correlation instead of linear regression. Sequences originating from chloroplasts or mitochondria were removed. Following these steps, no sequences classified as archaea were present in the final dataset. A total of 5.14 × 10^6^ out of 7.11 × 10^6^ reads (70.84 ± 11.49%) were retained after processing, generating 26 397 bacterial ASVs in total with an average of 47 908 ± 16 966 reads per sample. Rarefaction curves (Fig. S3) and Good’s coverage values, which were all above 99.86%, confirmed that the sequencing depth was sufficient to capture the bacterial diversity of each sample. Sequences were submitted to NCBI GenBank sequence read archive (SRA) under the accession number PRJNA1191203 (SUB15391150).

### Cultivation and characterization of 3GCR enterobacteria

Cultivation of 3GCR enterobacteria was performed from stained soil, rhizosphere, and phyllosphere suspensions. They were stored at −20°C in BPW supplemented with 20% (v/v) glycerol and transported from Mexico to Germany, which may have influenced the recovery of some cells. Selective pre-enrichment cultivation was performed in sterile 2 ml safe-lock tubes by adding 100 µl of each suspension to 900 µl *Escherichia coli* broth (EC broth, Sigma–Aldrich) containing 2 µg/ml cefotaxime (CTX, Sigma–Aldrich), followed by incubation in the dark for 24 h at 37°C on a horizontal shaker (150 rpm). Subsequently, 10 µl of the pre-enrichment cultures were streaked on Tryptone Bile X-Glucuronide Agar (TBX, Carl Roth GmbH, Germany) supplemented with 1 µg/ml CTX (TBX-CTX agar) and incubated for 3 h at 37°C, followed by 21 h at 44°C. Blue colonies (presumptive 3GCR *E. coli*) and beige colonies (other presumptive 3GCR enterobacteria) were selected and purified by streaking onto Müller–Hinton agar (MH, Carl Roth GmbH) containing 1 µg/ml CTX, followed by incubation at 37°C for 24 h. For long preservation, two loops of fresh pure bacterial cultures were resuspended in 500 µl newborn calf serum albumin (ThermoFisher Scientific, USA) and stored at −20°C. For molecular analysis, a loop of fresh bacterial cultures was suspended in 500 µl PCR-grade water (Carl Roth GmbH), and cell lysates were prepared by three freeze (−20°C) and heat cycles (100°C, 1 min 30 s). *Escherichia coli* strains were identified by diagnostic PCR detecting the *gadAB* and *uidA* genes (McDaniels et al. 1998, Doumith et al. 2012). All strains were compared by BOX-PCR fingerprints (Versalovic et al. 1994) and screened for extended-spectrum beta-lactamase (ESBL) genes (*bla*_CTX-M_, *bla*_TEM_, and *bla*_SHV_) using a multiplex-PCR system (Monstein et al. 2007). For phylogenetic identification, the 16S rRNA gene was amplified using primers EUB9f and EUB1492r (Lane 1991) and sequenced by Sanger Sequencing. PCR product purification and sequencing were performed by LGC Genomics (LGC Genomics GmbH, Germany) or Microsynth Seqlab GmbH (Germany). All primers and cycling conditions are listed in Supplementary Table S3. PCR reactions were performed in a total volume of 10 µl using 1 x DreamTaq buffer, 0.2 mM dNTPs (Thermo Scientific), 0.2 mg/ml bovine serum albumin (BSA), 0.02 U/µl DreamTaq DNA polymerase, and 1 µl of cell lysates as template. The size of the PCR products was verified by agarose gel electrophoresis and ethidium bromide staining. A DNA length marker (100 bp DNA ladder) was loaded in parallel. Reference strains as positive controls were used for each primer system in each PCR run. For BOX-PCR, different conditions were applied as described previously (Schauss et al. 2015). BOX-PCR fingerprinting patterns were analyzed using BioNumerics version 8 (Applied Maths, Belgium) according to Schauss et al. (2015). Strains with identical BOX-PCR patterns were assigned to the same genotype. Representative strains of each genotype were selected for partial 16S rRNA gene sequencing. Sequences were manually curated based on electropherograms using the Molecular Evolutionary Genetics Analysis (MEGA) program version 12 (Kumar et al. 2024). Closest related type strains were identified using the Basic Local Alignment Search Tool (BLAST; https://blast.ncbi.nlm.nih.gov/Blast.cgi) against the NCBI RefSeq 16S rRNA gene sequence database. Phylotypes were defined based on a phylogenetic analysis and sequence similarity using ARB (http://www.arb-home.de/) as described in Franco et al. (2020). All sequences generated in this study were deposited in GenBank under accession numbers PV492330 to PV492401. The 16S rRNA gene sequences of the bacterial strains were used for comparative analysis with ASV sequences using MEGA 12.

### Data analysis and statistics

Statistical analysis was performed in R (R Core Team 2021; v.4.2.1) using the “tidyverse” set of packages (Wickham et al. 2019⁠), “ggplot2” (Wickham 2016), “dplyr” (Wickham et al. 2023), “reshape2” (Wickham 2007), “stringr” (Wickham 2023), “vegan” (Oksanen et al. 2024), “dream” (Hoffman and Roussos 2021), and phyloseq (McMurdie and Holmes 2013). Euclidean distances of ARG and MGE relative abundances were used for PCA ordinations. Significant differences (*P* < 0.05) in 16S rRNA gene copies, ARG and MGE absolute and relative abundances between UWW and TWW were assessed using the Wilcoxon test, while differences among treatments in soil, rhizosphere, and phyllosphere were evaluated by linear regression with bootstrap values.

Amplicon sequencing data were analyzed in rarefied abundance (30 203 sequences per sample). Bray-Curtis distances of rarefied data were used to estimate β-diversity, and Permutational Multivariate Analysis of Variance (PERMANOVA; vegan package) to assess the effects of water quality (water type and spiking level) and soil type on soil, rhizosphere, and phyllosphere bacterial communities. Significant differences (*P* < 0.05) in the relative abundance of the most abundant bacterial taxa were assessed using the Wilcoxon test for WW samples and Dunn’s test with Benjamini–Hochberg correction for stained soil, rhizosphere, and phyllosphere samples. Mantel tests evaluated correlations between distance matrices, and differential abundance was tested via logistic regression (glm) with Benjamini–Hochberg correction in R.

## Results

### Slight reduction of ARGs and MGEs after WW treatment

**Figure 1:**
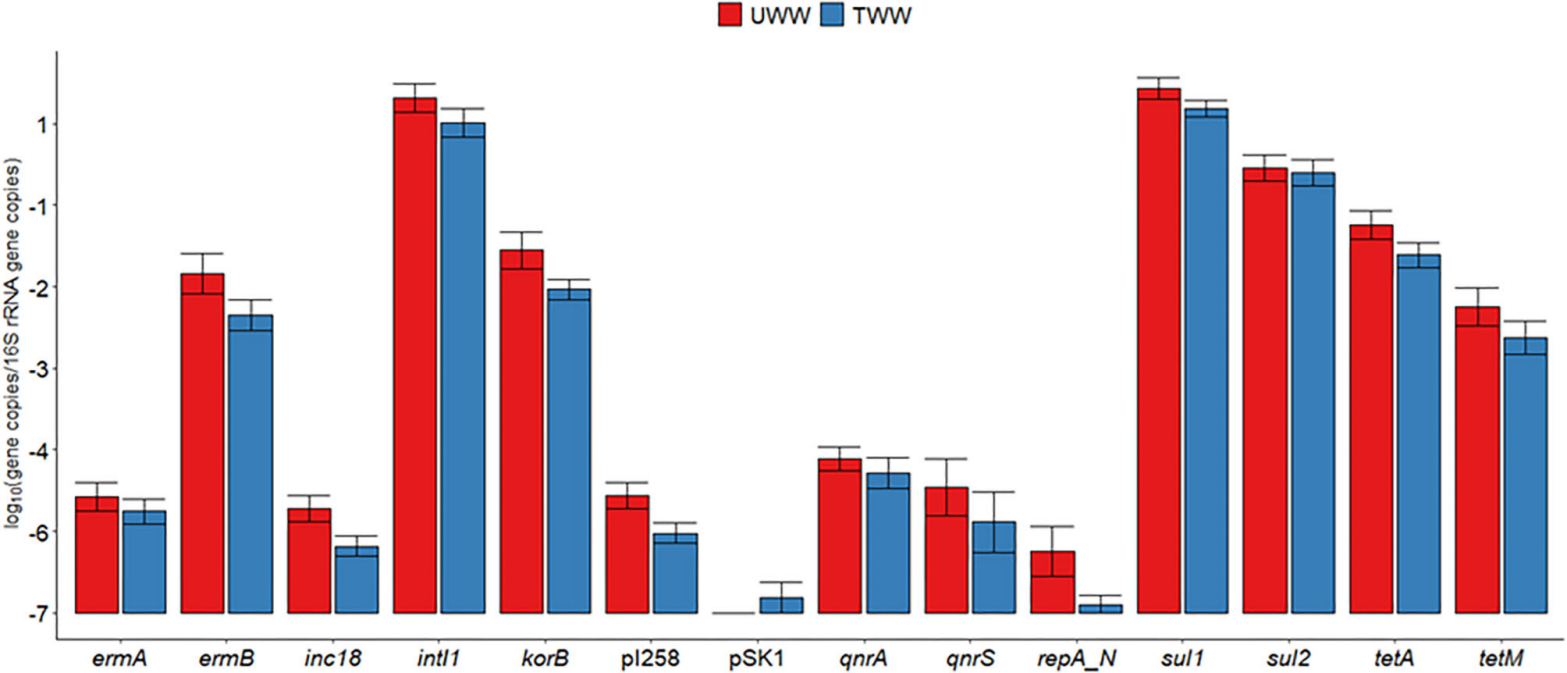
ARG and MGE relative abundances (log_10_ copies per 16S rRNA gene copies) in UWW and TWW samples. No significant differences observed (*P* > 0.05; Wilcoxon test).

COD and TOC values were lower in TWW than in UWW, whereas the concentrations of several antibiotics and total dissolved nitrogen showed no reduction or were even higher after WW treatment (Table 1). Bacterial 16S rRNA gene copy numbers ranged from 8.32 to 9.88 log_10_ gene copies/L across the different water samples (Fig. S4). All ARGs and MGEs were detected in all WW samples, except for the *Staphylococcus* pSK1 plasmid, which was found only in one TWW sample (Fig. S4). The highest relative abundances were observed for *sul1* and *intI1* genes (Fig. 1), while *sul2, tetA, tetM* genes, IncP-1 plasmids, and *ermB* genes were detected at lower relative abundances (Fig. 1). Unexpectedly, ARG and MGE distribution patterns showed no statistically significant differences between UWW and TWW samples (Fig. S5; PERMANOVA, R^2^ = 0.20, *P* = 0.105).

### Irrigation with spiked WW significantly influenced ARG and MGE profiles in soil but not in rhizosphere or phyllosphere

In soil, bacterial 16S rRNA gene copy numbers were slightly higher in samples from stained soil (10.97± 0.31 log_10_ 16S rRNA gene copies per g dry soil) compared to unstained soil (10.80 ± 0.16 log_10_ 16S rRNA gene copies per g dry soil). However, this difference was not significant (*P* > 0.05; linear regression with bootstrap values) (Fig. S6). Irrigation with different water qualities also did not significantly affect 16S rRNA gene copy numbers per gram of dry soil (Fig. S6; *P* > 0.05; linear regression with bootstrap values).

**Figure 2:**
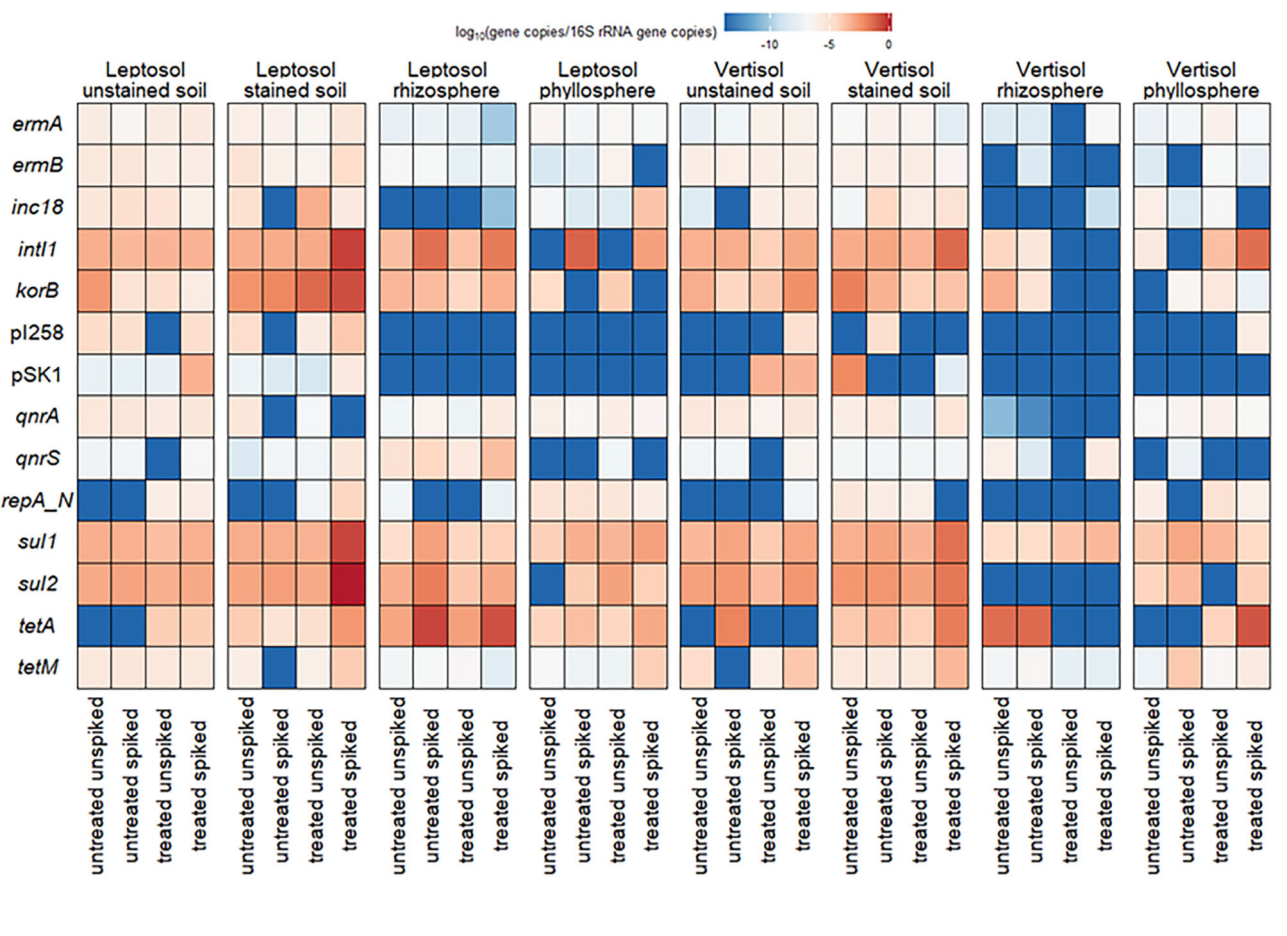
ARG and MGE relative abundances (log_10_ gene copies/16S rRNA gene copies) in samples from unstained soil, preferential water flow path soil (stained soil), cilantro rhizosphere, and phyllosphere irrigated with UWW or TWW, both unspiked and spiked with antibiotics and disinfectants.

**Figure 3:**
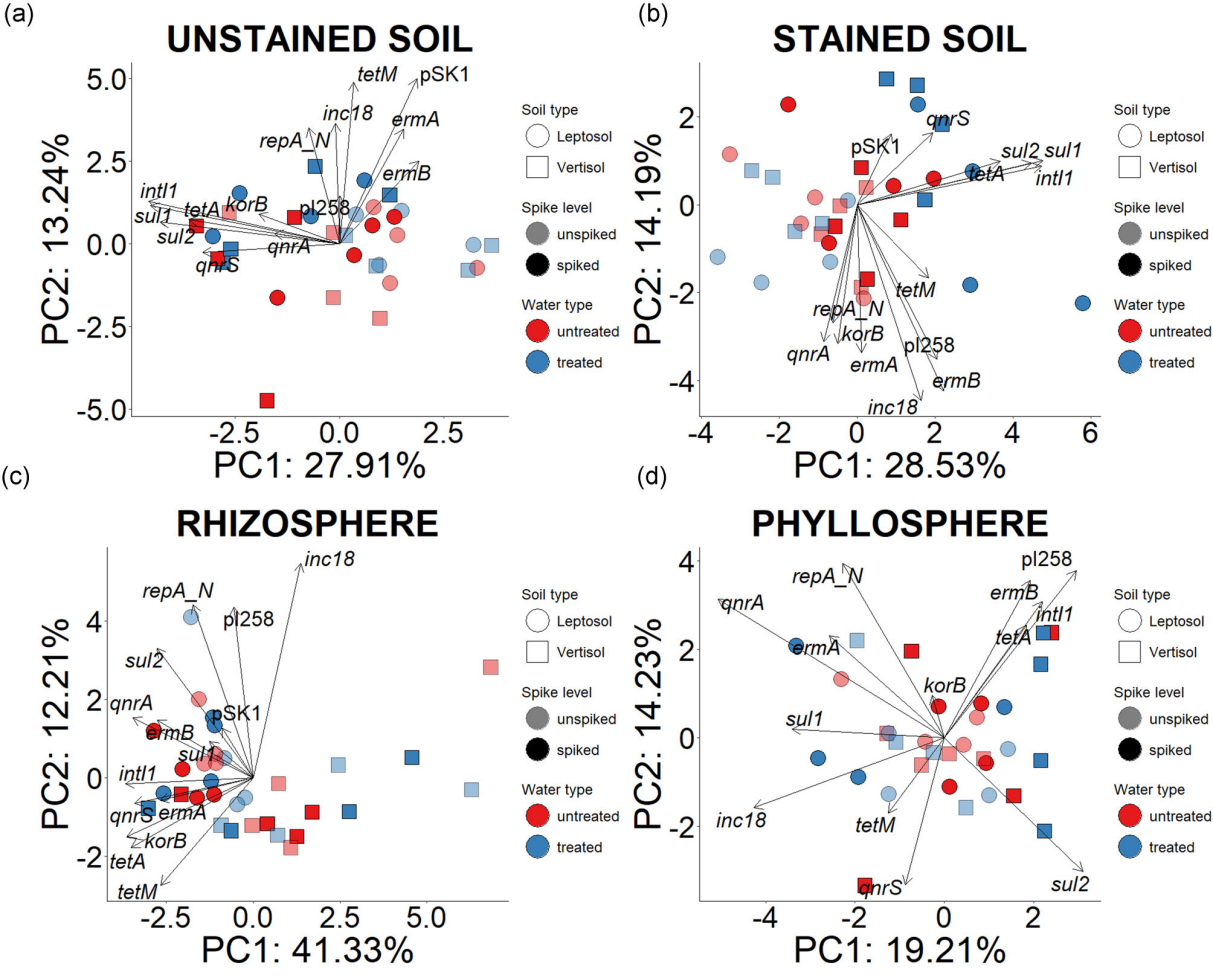
Principal Component Analysis of ARG and MGE distributions in (A) Leptosol and Vertisol soils from unstained soil, (B) Leptosol and Vertisol soils from preferential water flow path soil (stained soil), (C) cilantro rhizosphere, and (D) phyllosphere samples irrigated with UWW or TWW, both unspiked and spiked with antibiotics and disinfectants. Significance of separation was assessed with Global PERMANOVA test (A: spike level: R^2^ = 0.12, *P* < 0.05; soil type: R^2^ = 0.03, *P* > 0.05; water type: R^2^ = 0.04, *P* > 0.05; B: spike level: R^2^ = 0.11, *P* < 0.05; soil type: R^2^ = 0.03, *P* > 0.05; water type: R^2^ = 0.02, *P* > 0.05; C: spike level: R^2^ = 0.03, *P* > 0.05; soil type: R^2^ = 0.38, *P* < 0.05; water type: R^2^ = 0.02, *P* > 0.05; D: spike level: R^2^ = 0.05, *P* > 0.05; soil type: R^2^ = 0.018, *P* > 0.05; water type: R^2^ = 0.02, *P* > 0.05).

All ARGs and MGEs were detected across all soil samples, with varying relative abundances within the same treatment, likely due to the use of intact soil monolith cores rather than homogenized soil. The highest relative abundances in soil were observed for *tetA, sul1, sul2*, and *intI1* genes, followed by IncP-1 plasmids, *ermB*, and *ermA* genes (Fig. 2 and Fig. S7A-D). ARG and MGE profiles were significantly distinct between stained and unstained soil (Fig. S8; PERMANOVA, R^2^ = 0.06, *P* < 0.05), with a significant influence of the spiking of the irrigation water (Fig. S8; PERMANOVA, R^2^ = 0.09, *P* < 0.05, Fig. 3A; PERMANOVA, R^2^ = 0.12, *P* < 0.05, and Fig. 3B, R^2^ = 0.11, *P* < 0.05). Specifically, samples from stained soil showed higher relative abundance of *sul1* and *sul2, intI1* genes, IncP-1, and *Enterococcus* plasmids compared to samples from unstained soil. Differences were significant for IncP-1 and *Enterococcus* plasmids (*P* < 0.05 and *P* < 0.05, respectively, linear regression with bootstrap values, Table S4) (Fig. 2 and Fig. S7A-D). Irrigation with spiked WW, either UWW or TWW, significantly increased the relative abundances of *intI1, sul1, sul2*, and *tetA* genes in all soil compartments (*P* < 0.05, linear regression with bootstrap values, Table S5) (Fig. 2 and Fig. S7A-D).

In the cilantro rhizosphere, the highest relative abundances of ARGs and MGEs were found in Leptosol soils for *tetA, intI1*, and *sul2* genes (Fig. 2 and Fig. S7E-F). IncP-1 plasmids, *sul1, qnrA*, and *qnrS* genes were also found in high relative abundances in the rhizosphere of both soil types. ARG and MGE profiles in the rhizosphere were only influenced by the soil type (Fig. 3C; PERMANOVA, R² = 0.38, *P* < 0.05) with Leptosol columns exhibiting higher relative abundances of most ARGs and MGEs compared to Vertisol columns (Fig. 2 and Fig. S7E-F). Spiked WW irrigation significantly increased the relative abundance of *tetA* (*P* < 0.05, linear regression with bootstrap), *intI1* (*P* < 0.05, linear regression with bootstrap), and *sul2* genes (*P* < 0.05, linear regression with bootstrap values) in the rhizosphere of both soil types (Fig. 2 and Fig. S7E-F, Table S5).

In the phyllosphere, the highest relative abundance of ARGs across the two soil types was observed for *tetA, sul2*, and *sul1* genes, although high variability was found among the different samples (Fig. 2 and Fig. S7G-H). Irrigation with spiked WW did not affect the overall ARG and MGE distribution patterns in the phyllosphere compared to unspiked WW (Fig. 3D; PERMANOVA, R² = 0.05, *P* > 0.05). However, spiked WW irrigation led to a significant increase in the relative abundance of specific ARGs, namely *ermA* (*P* < 0.05, linear regression with bootstrap values) and *qnrA* (*P* < 0.05, linear regression with bootstrap values) in cilantro phyllosphere of Leptosol samples irrigated with TWW and Vertisol samples irrigated with UWW (Fig. S7G-H, Table S5).

### Distinct bacterial communities in UWW *vs*. TWW

First, the bacterial community composition of the different irrigation water types was characterized. Bacterial community compositions differed significantly between UWW and TWW samples (Fig. S9; PERMANOVA, R² = 0.36, *P* < 0.05). The most prevalent bacterial phyla identified in both UWW and TWW samples were *Pseudomonadota* (formerly *Proteobacteria*) (dominated by *Gammaproteobacteria* and *Alphaproteobacteria*), *Bacteroidota* (represented by *Bacteroidia*), *Actinomycetota* (formerly *Actinobacteriota*) [mainly represented by *Actinomycetia* (formerly *Actinobacteria*)], followed by *Bacillota* (formerly *Firmicutes*; predominantly *Clostridia*), and *Verrucomicrobiota* (Fig. S10A-B). WW treatment significantly increased the relative abundance of *Pseudomonadota* (*P* <0.05; Wilcoxon test) while the relative abundance of *Actinomycetota* decreased (*P* < 0.05; Wilcoxon test). *Nitrosomonas, Romboutsia, Simplicispira, Sediminibacterium, Leucobacter, Pedobacter, Reyranella, Acidovorax*, and *Microbacterium* were among the 15 most abundant ASVs (Fig. S11). Among these, *Leucobacter*, an unclassified *Alcaligenaceae, Acidovorax*, and *Microbacterium* showed significantly higher relative abundances in UWW compared to TWW. In contrast, an unclassified *Rhizobiales, Simplicispira* (ASV135 and ASV159), and *Reyranella* had significantly higher relative abundances in TWW compared to UWW (*P* < 0.05; Wilcoxon test) (Fig. S11).

### Stained soil and rhizosphere bacterial communities were primarily influenced by the soil type but also by the spiking of the irrigation water

**Figure 4:**
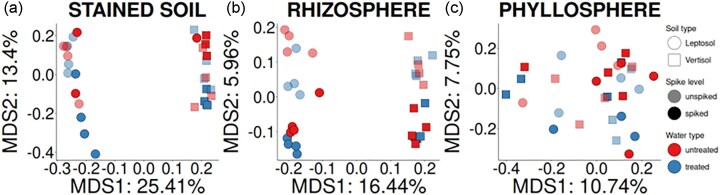
Multidimensional scaling analysis of bacterial community structure based on Bray–Curtis dissimilarities from 16S rRNA gene amplicon sequencing data in (A) samples from preferential water flow path soil (stained soil), (B) rhizosphere, and (C) phyllosphere irrigated with UWW or TWW, both unspiked and spiked with antibiotics and disinfectants. Significance of separation was assessed with Global PERMANOVA test. (A: soil type: R^2^ = 0.23, *P* < 0.05; spike level: R^2^ = 0.05, *P* < 0.05; water type: R^2^ = 0.04, *P* > 0.05; B: soil type: R^2^ = 0.16, *P* < 0.05; spike level: R^2^ = 0.06, *P* < 0.05; water type: R^2^ = 0.05, *P* < 0.05; C: soil type: R^2^ = 0.03, *P* > 0.05; spike level: R^2^ = 0.03, *P* > 0.05; water type: R^2^ = 0.04, *P* > 0.05).

Given the distinct ARG and MGE profiles and higher relative abundances in stained than in unstained soils, bacterial community composition was analyzed only in soils exposed to irrigation water percolating via preferential flow paths (stained soil), rhizosphere, and phyllosphere. In stained soil samples, the bacterial communities were primarily shaped by soil type (Fig. 4A; PERMANOVA, R² = 0.23, *P* < 0.05), although the spiking level also had a significant influence (Fig. 4A, R² = 0.05, *P* < 0.05). Unexpectedly, the type of irrigation water did not have a significant effect (Fig. 4A). ARG/MGE profiles were weakly but significantly correlated with Bray–Curtis distances of bacterial communities (Mantel *rho* = 0.2595, *P* < 0.05). *Pseudomonadota* and *Actinomycetota* exhibited the highest relative abundance across all soil samples. Their relative abundance ranged from 18% to 60% across both Leptosol and Vertisol samples. The phyla *Chloroflexi, Acidobacteriota*, and *Bacteroidota* were present at lower relative abundances (below 16%) (Fig. S12A). *Methylophilus, Nocardioides, Methylotenera, Gaiella*, and *Intrasporangium* were among the most abundant genera in stained soil (Fig. S13A).

Rhizosphere bacterial communities were influenced by the soil type (Fig. 4B; PERMANOVA, R² = 0.16, *P* < 0.05), and to a lower degree, by the spiking of the irrigation water (Fig. 4B, R² = 0.06, *P* < 0.05). Water type (Fig. 4B, R² = 0.05, *P* < 0.05) had a modest yet statistically significant effect. However, the correlations between ARG/MGE profiles and rhizosphere bacterial community Bray–Curtis distances were not significant (Mantel *rho* = 0.029, *P* = 0.372). *Pseudomonadota* dominated rhizosphere samples (relative abundance of 83% ± 7%), while *Actinomycetota, Bacteroidota, Chloroflexi*, and *Bacillota* were less abundant (<1%–23%) (Fig. S12B). *Pseudomonas* was the dominant genus in the cilantro rhizosphere, displaying significantly higher relative abundance in the rhizosphere samples from Leptosols (49% ± 9%) compared to Vertisols (40% ± 9%) (*P* < 0.05; Wilcoxon test with Benjamini–Hochberg adjustment) (Fig. S13B).

In contrast to the rhizosphere and stained soil, phyllosphere bacterial communities showed no significant response to the soil type, spiking of the irrigation water or water type (soil type: R² = 0.03, *P* > 0.06; spike level: R² = 0.03, *P >* 0.06; water type: R² = 0.04, *P >* 0.06; Fig. 4C). These results were confirmed by the correlation between ARG/MGE profiles and the Bray–Curtis distances from phyllosphere bacterial communities, which was negative and non-significant (Mantel *rho* = −0.110, *P* > 0.06). The phyllosphere was dominated by *Pseudomonadota* (64% ± 22%) and *Bacillota* (34% ± 21%) (Fig. S12C) with *Exiguobacterium, Pseudomonas, Pantoea*, and *Acinetobacter* among the most abundant genera (Fig. S13C).

**Figure 5:**
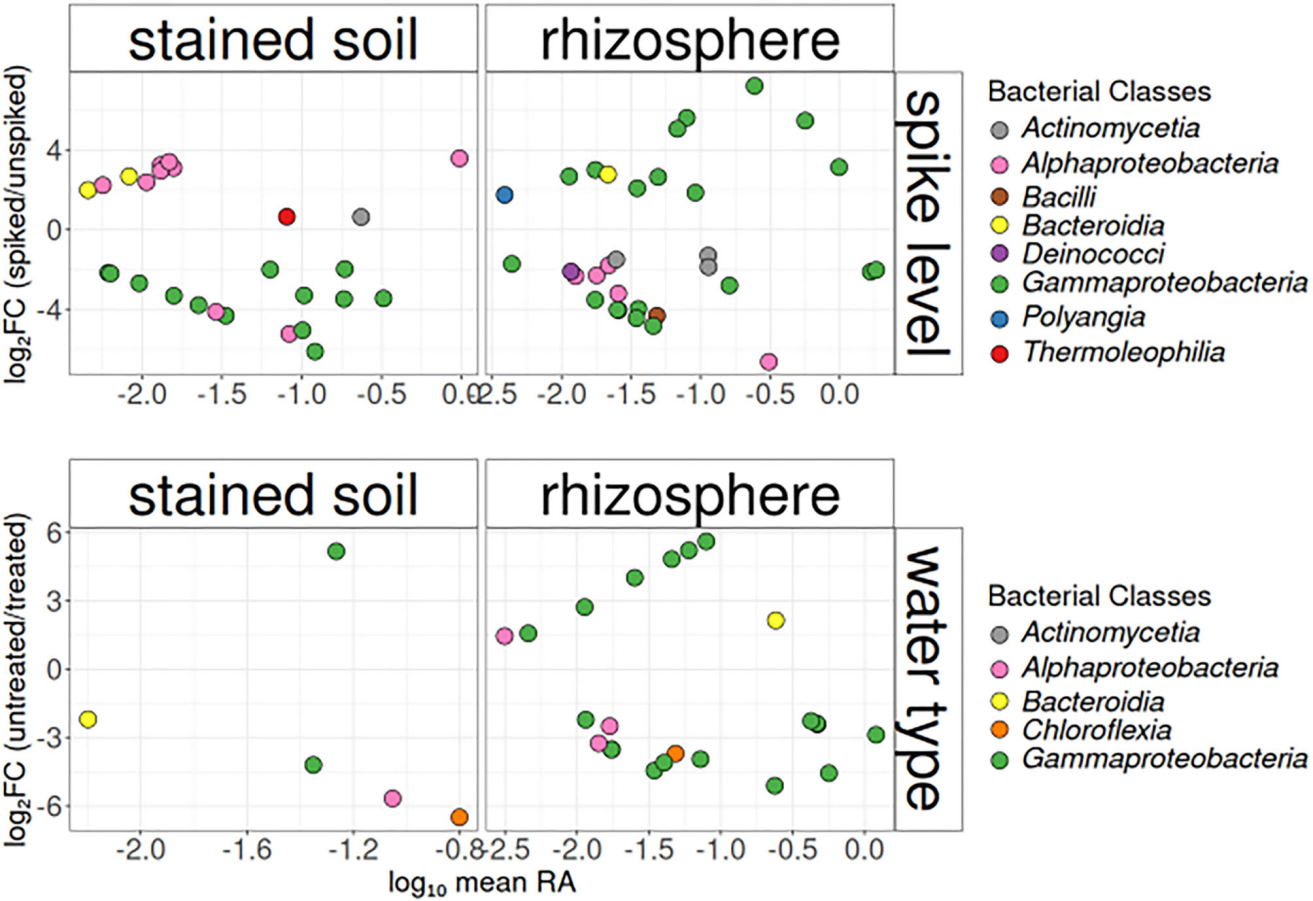
Differentially abundant ASVs from preferential water flow path soil and rhizosphere irrigated with UWW or TWW, both unspiked and spiked with antibiotics and disinfectants. The *x*-axis shows the log₁₀-transformed average of their relative abundance (log₁₀ RA), and the *y*-axis shows the log₂-transformed fold change (log₂ FC) in abundance between two conditions.

To investigate whether the spiking of the irrigation water and water type influenced the relative abundance of specific ASVs from stained soil, rhizosphere, and phyllosphere, a logistic linear regression analysis was conducted (Fig. 5 and Table S6).

In stained soils, the relative abundance of ASVs affiliated with the class *Alphaproteobacteria*—six from the order *Rhizobiales*, one from *Caulobacterales*, two from the phylum *Actinomycetota* (one *Actinomycetia* and one *Thermoleophilia*), and two from the class *Bacteroidia* (order *Flavobacteriales*)—was significantly higher in soil columns irrigated with spiked WW compared to those irrigated with unspiked WW. Conversely, multiple ASVs within *Gammaproteobacteria*—notably *Methylibium, Methylotenera* (family *Methylophilaceae*), and other *Burkholderiales*, as well as ASVs from *Xanthomonadales, Diplorickettsiales*, and two *Alphaproteobacterial* ASVs—*Azospirillum* and *Hyphomicrobium*, were less abundant under spiked WW irrigation (Fig. 5 and Table S6).

With respect to the water type, only one ASV assigned to *Methylotenera* (*Gammaproteobacteria*) showed a significantly higher relative abundance in soil columns irrigated with UWW compared to TWW, while the relative abundances of several ASVs within the *Gammaproteobacteria, Bacteroidia, Chloroflexia*, and *Alphaproteobacteria* were significantly lower in UWW-irrigated columns (Fig. 5 and Table S6).

In rhizosphere samples, several ASVs affiliated with the class *Gammaproteobacteria* were differentially affected by spiked WW irrigation. Notably, within the order *Enterobacterales*, three ASVs (assigned to *Citrobacter* and *Rheinheimera*) showed significantly higher relative abundances after irrigation with spiked WW compared to unspiked WW. Within the order *Pseudomonadales*, two ASVs (assigned to *Pseudomonas*) showed significantly higher relative abundances. Several ASVs within the order *Burkholderiales* also increased following irrigation with spiked WW. Beyond *Gammaproteobacteria*, significantly higher relative abundances were also observed for ASVs affiliated with *Bacteroidia* and *Polyangia*. By contrast, ASVs from the class *Alphaproteobacteria, Actinomycetia, Deinococci*, and *Bacilli* exhibited significantly lower relative abundances under spiked WW irrigation (Fig. 5 and Table S6).

Regarding the effect of the water type on rhizosphere taxa, a similar trend was observed. Several gammaproteobacterial ASVs showed significantly higher relative abundances in rhizosphere samples irrigated with UWW compared to samples irrigated with TWW. ASVs belonging to the *Enterobacterales* were significantly more abundant after irrigation with UWW compared to TWW. In addition, several ASVs affiliated with the order *Burkholderiales* and *Xanthomonadales*, and one ASV belonging to the class *Bacteroidia* displayed significantly higher relative abundances in the rhizosphere irrigated with UWW compared to TWW. Conversely, three ASVs affiliated with the order *Pseudomonadales* within the *Gammaproteobacteria*, one ASV belonging to the *Actinomycetia* and one ASV within the *Chloroflexia* exhibited significantly lower relative abundances after irrigation with UWW compared to TWW (Fig. 5 and Table S6).

In phyllosphere samples, no ASVs significantly responded to the different water qualities. However, one ASV affiliated with the genus *Massilia*, belonging to the order *Burkholderiales*, showed a significantly higher abundance in the phyllosphere of cilantro plants grown in Vertisol soils compared to Leptosol soils (Table S6).

## 3GCR *Citrobacter* were predominantly isolated from TWW-irrigated stained soils and rhizosphere

**Figure 6:**
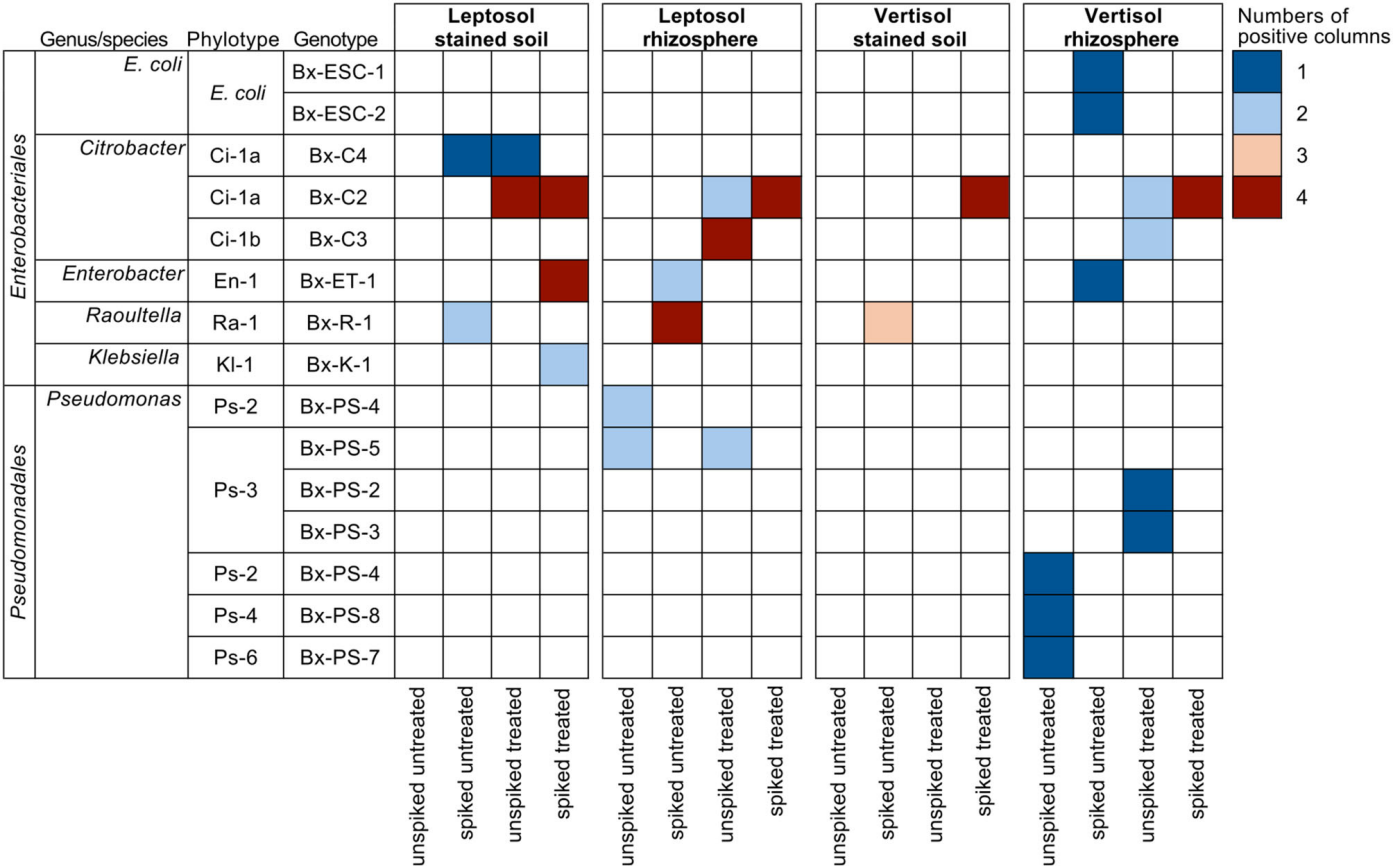
3GCR enterobacterial isolates from stained soil and rhizosphere samples irrigated with UWW or TWW, both spiked and unspiked with antibiotics and disinfectants. No 3GCR enterobacteria were isolated from phyllosphere samples. Four soil columns per treatment were analyzed. Phylotype identification is based on partial 16S rRNA gene sequencing, phylogenetic analysis (for details see Suppl. Fig. 1) and PCR for *E. coli* identification. Genotypes were defined by genomic fingerpringting using BOX-PCR.

3GCR enterobacteria were isolated from enrichments of stained soil and rhizosphere, but not from phyllosphere samples. Total 119 colonies were randomly selected for further characterization. Among those, 91% (n = 108) represented 3GCR enterobacteria. They were assigned based on 16S rRNA gene sequencing to the genera *Citrobacter* (76%; n = 90), *Enterobacter* (6%; n = 7), *Raoultella* 5% (n = 6), *Klebsiella* (3%; n = 3), and to *E. coli* (2%; n = 2) (Fig. 6; Fig. S14; Table S7). The remaining 9% (n = 11) were assigned to the genus *Pseudomonas*. The characterization of all 3GCR *Citrobacter* strains by BOX-PCR fingerprinting and 16S rRNA gene sequencing revealed three different BOX-PCR genotypes of two different phylotypes (16S rRNA gene sequence-based clusters). By far the most abundant genotype was Ci-1a_Bx-C2 (n = 76). All strains from Ci-1a_Bx-C2 originated only from TWW-irrigated stained soils of both soil types and corresponding rhizosphere samples. (Fig. S15A). The *Citrobacter* genotype Ci-1a_Bx-C4 (n = 8) was isolated from stained soil of two Leptosol columns irrigated with spiked UWW and unspiked TWW (Fig. 6). Both *Citrobacter* genotypes were assigned to the *C. freundii* complex (Fig. S14, Table S7). The third *Citrobacter* genotype (Ci-1b_Bx-C3; n = 6) was obtained exclusively from the rhizosphere of cilantro plants grown in both soil types and irrigated with unspiked TWW (Fig. 6). 3GCR *Enterobacter* and *Raoultella* strains were isolated from stained soil and rhizosphere samples of Leptosol and Vertisol columns irrigated with spiked UWW or spiked TWW, while the 3GCR *Klebsiella* strains were isolated only from stained soil of Leptosol columns irrigated with spiked TWW (Fig. 6). All *Enterobacter, Raoutella*, and *Klebsiella* strains shared identical BOX-PCR fingerprint patterns within the respective genus (Fig. S15B). Two *E. coli* strains with distinct BOX-PCR patterns were isolated from rhizosphere samples of Vertisol columns irrigated with spiked UWW (Fig. 6, S15 B). Most enterobacterial strains (106 of 108) carried a *bla*_CTX-M_ and *bla*_TEM_ gene (Fig. S15A-B, Table S7). Two *Klebsiella* sp. strains carried in addition a *bla*_SHV_ gene and one *E. coli* strain carried only *bla*_CTX-M_ together with *bla*_SHV_.

ASVs corresponding to the cultivated enterobacterial genera (*Citrobacter, Raoultella, Enterobacter*, and *Klebsiella*) were detected only in rhizosphere samples (Table S8). The 16S rRNA gene fragments sequences showed complete coverage relative to the ASV sequences. Seven *Citrobacter* ASVs were detected in all rhizosphere samples. The *Citrobacter* strains from genotypes Ci-1a_Bx-C2 and Ci-1a_Bx-C4 shared 100% identical 16S rRNA gene sequences with two of the seven detected *Citrobacter* ASVs. These two ASVs were also detected at low read abundances in two of the five analyzed TWW samples but not in UWW samples (Table S8).

## Discussion

The study of plant-associated and soil microbiomes has attracted increasing scientific attention due to concerns that agricultural environments—particularly those irrigated with WW—may serve as reservoirs and transmission pathways for ARB, ARGs, and MGEs (Larsson and Flach 2021). The co-occurrence of antibiotics, heavy metals, and disinfectants—alongside ARB harbouring ARGs—may favour ARG dissemination through MGEs such as plasmids as well as integrons in both bulk soil and rhizosphere, potentially enabling the colonization of edible plant tissues by pathogens and bacteria carrying these elements (Becerra-Castro et al. 2015, Ye et al. 2016, Alegbeleye et al. 2018, 2019b,c, Cerqueira et al. 2019a, Piña et al. 2020). In this context, by using undisturbed monolithic soil columns, our study evaluated the impact of WW irrigation and the role of the soil type on the relative abundances and diversity of ARGs and MGEs, as well as on the bacterial community in soils—including preferential flow path soil—and plant-associated microbiomes (rhizosphere and phyllosphere), key hotspots for pollutant transport and ARG dissemination.

Analysis of UWW and TWW samples showed a noticeable decrease in COD and TOC following treatment. In contrast, the concentrations of several antibiotics and total dissolved nitrogen remained unchanged or were even higher after WW treatment. The latter two phenomena are mainly attributed to the transformation of pharmaceutical metabolites—excreted by humans—back into their original forms during WW treatment (Kumar et al. 2022), and to the mineralization of particulate organic N in UWW that was not captured by the analysis of N_tot_, since only dissolved nitrogen was measured. Bacterial 16S rRNA gene copy numbers in UWW and TWW samples were comparable, with only slightly lower levels in TWW. A similar observation was reported in our previous study from the same WWTP (Soufi et al. 2025) and has also been documented in Oliveira et al. (2016), Rafraf et al. (2016), and Rodríguez et al. (2021). The minor reduction in bacterial 16S rRNA gene copy numbers between the UWW and TWW can be attributed to the Atotonilco WWTP’s strategy of maintaining nutrients in the TWW, which is beneficial for local agricultural use (Garduño-Jiménez et al. 2023). All WW samples harboured the investigated ARGs and MGEs, except the *Staphylococcus* pSK1 resistance plasmid. The highest relative abundances in both UWW and TWW were found for *sul1*, followed by the *intI1* gene, consistent with earlier reports (Soufi et al. 2025). This suggests that the co-occurrence of WW-borne pollutants favours the spread of ARGs associated with integrons that are frequently carried on plasmids (Heuer et al. 2011, Heuer and Smalla 2012, Berendonk et al. 2015, Chen et al. 2015, Gillings et al. 2015, Haenelt et al. 2023, Shintani et al. 2023, Hauschild et al. 2025). TWW showed only a slight reduction (ranging from 0.06 to 1.58 orders of magnitude) in ARG and MGE relative abundance levels compared to UWW, highlighting the limited effectiveness of conventional treatment in removing bacteria carrying these elements, as reported also by Rafraf et al. (2016) and Rodríguez et al. (2021). The most abundant phylum in both UWW and TWW was *Pseudomonadota*, with a significantly higher relative abundance in TWW, aligning with our previous study by Soufi et al. (2025), followed by *Bacteroidota* and *Actinomycetota*, as also reported by Azli et al. (2022), Li et al. (2023), and Lira et al. (2020). It should be noted that some changes in the chemical and microbial composition of the stored WW may have occurred over time. However, such a change is expected under storage conditions in practice and reflects realistic irrigation scenarios (Iakovides et al. 2025).

In soils, bacterial 16S rRNA gene copy numbers were slightly higher in stained soil exposed to water percolating via preferential flow paths compared to unstained soil, likely due to nutrients and organic matter introduced with WW (Becerra-Castro et al. 2015, Mola et al. 2024). However, the bacterial 16S rRNA gene copy numbers were lower after irrigation with spiked WW, possibly due to the antimicrobial effects of the spiked substances (Cleary et al. 2016, Markowicz et al. 2021, Katsivelou et al. 2023). ARG and MGE profiles differed significantly between stained and unstained soils, with stained soils exhibiting higher ARG abundances. This aligns with previous findings suggesting that flow paths can serve as hotspots for the occurrence and accumulation of antibiotics and resistance genes in soil (Heyde et al, under revision, Lüneberg et al. 2018a). Spiking the irrigation water significantly increased ARG and MGE relative abundances across stained and unstained soils, supporting prior research indicating that prolonged WW irrigation promotes ARG enrichment via selective pressure exerted by persistent micropollutants (Jechalke et al. 2015, Kampouris et al. 2021, Franklin et al. 2024).

Additional analysis of the soil bacterial community composition in stained soil revealed that the soil type had the strongest effect, in agreement with our previous findings (Soufi et al. 2025) and other reports (Krause et al. 2020, Obayomi et al. 2021, Mola et al. 2024). Furthermore, the irrigation with spiked water caused a modest but significant effect on the bacterial community, supported by the Mantel test, which revealed a weak yet significant correlation between soil bacterial community composition and ARG/MGE profiles. This effect was also observed by Soufi et al. (2025), suggesting that shifts in ARG and MGE profiles may be caused by changes in the soil bacterial community composition, underscoring the importance of sampling preferential water flow path soil for their detection (Reichenberger et al. 2002, Kim et al. 2008, Villamizar and Brown 2017, Lüneberg et al. 2018a, Salazar-Ledesma et al. 2018, Zhang Yinghu et al. 2018). In contrast, the irrigation with UWW or TWW had no significant impact on the overall soil bacterial community composition assessed by 16S rRNA gene sequencing. This contrasts with studies reporting pronounced microbial shifts due to irrigation water quality (Onalenna and Rahube 2022), but is in line with studies indicating that soil microbial communities may exhibit resilience to variations in irrigation water quality (Becerra-Castro et al. 2015, Lüneberg et al. 2018b). *Pseudomonadota* and *Actinomycetota* were the most abundant soil bacterial phyla, in agreement with previous reports on their prevalence in WW-irrigated environments (Ibekwe et al. 2018, Xu et al. 2020, Gallego et al. 2021). *Pseudomonadota* are known for their role in spreading ARGs and MGEs (Hu et al. 2016), while *Actinomycetota* are recognized for their capacity to produce a wide array of secondary metabolites and for harbouring ARGs (D’Costa et al. 2011).

The effects of spiking UWW or TWW were further assessed for bacterial ASVs significantly responding to exposure to WW in stained soils. Some ASVs affiliated to *Rhizobiales, Actinomycetia*, and *Bacteroidia* were significantly more abundant in stained soil irrigated with spiked WW compared to those irrigated with unspiked WW. These taxa potentially exhibit high metabolic versatility in degrading organic pollutants and tolerance to chemical stressors (Teng et al. 2015, Zhang et al. 2017, Izabel-Shen et al. 2022, Wang et al. 2022, Behera and Das 2023, Porras-Socias et al. 2024).

Irrigation with UWW led to a significantly higher relative abundance of an ASV affiliated with *Methylotenera* in stained soils compared to TWW. In our previous study (Soufi et al. 2025), two *Methylotenera* ASVs—among the most abundant in both WW and soil—also increased following WW irrigation, suggesting they were likely introduced via the irrigation water or benefitted from it. In contrast, four ASVs within the classes *Gammaproteobacteria, Bacteroidia, Chloroflexia*, and *Alphaproteobacteria*—affiliated with the genera *Pseudoxanthomonas, Chryseobacterium, Herpetosiphon*, and *Hyphomicrobium*, respectively—showed significantly lower relative abundances in stained soils after irrigation with UWW compared to TWW. Their lower relative abundances in soils irrigated with UWW than in those irrigated with TWW likely reflect competition by fast-growing, copiotrophic taxa favoured under nutrient-rich UWW conditions (Koch 2001).

In the cilantro rhizosphere, the soil type was the only driver of ARG and MGE profiles, with Leptosol (sandy loam) exhibiting higher ARG—particularly *tetA* and *sul2—*and *intI1* levels compared to Vertisol (clay). This is likely due to differences in soil texture and physicochemical properties influencing pollutant binding and sequestration, although Vertisols received more irrigation water and thus more spiked pollutants. Marano et al. (2019) reported positive correlations between *intI1* abundance in irrigation water and sandy loam soils, but not in clay-rich soils, supporting the hypothesis that soil characteristics strongly influence ARG distribution through differences in the microbial community composition and micropollutant bioavailability (Jechalke et al. 2014, Wang et al. 2020, Zhang et al. 2021, Song et al. 2024, Zeng et al. 2025). In our study, irrigation water type (UWW *vs*. TWW) did not influence the abundance of ARG and MGE in the cilantro rhizosphere, consistent with Cerqueira et al. (2019c). Nevertheless, spiking the irrigation water further increased the abundance of *tetA, sul2*, and *intI1* in the rhizosphere across both soil types, confirming that the presence of elevated concentrations of antibiotics and disinfectants in the environment promotes ARG and MGE abundance and diversity (Phan et al. 2024). Similarly, Shen et al. (2019) reported higher *intI1* occurrence in lettuce roots irrigated with pharmaceutical-contaminated water.

In our study, rhizosphere bacterial communities were predominantly shaped by soil type, with dominant phyla including *Pseudomonadota, Actinomycetota*, and *Bacteroidota*, including a high abundance of *Pseudomonas* sp. consistent with previous studies (Berg and Smalla 2009, Philippot et al. 2013, Schreiter et al. 2014, Zhang Yuping et al. 2018, Echeverry-Gallego et al. 2024, Kouadri 2024). Elevated *Pseudomonas* abundance in the rhizosphere may reflect high nutrient availability due to long-term WW irrigation (Zolti et al. 2019). Spiking the irrigation water and water type also influenced the rhizosphere bacterial communities, though to a lesser extent than the soil type. Water treatment was reported to contribute to microbial shifts in the root-associated microbiome of tomato and lettuce (Zolti et al. 2019). This aligns with reports showing that pharmaceuticals in irrigation water can alter root microbiomes, including changes following irrigation with WW or fertilizer solution spiked with pharmaceuticals (Shen et al. 2019, Cerqueira et al. 2020, Man et al. 2020, Bigott et al. 2022, Bhattacharjee et al. 2024, Phan et al. 2024).

Spiking the irrigation water caused a significant enrichment of several gammaproteobacterial ASVs—affiliated with *Citrobacter* and *Rheinheimera, Pseudomonas*, and members of the *Burkholderiales*, including *Achromobacter, Methylophilus, Variovorax, Acidovorax*, and *Pseudorhodoferax*—as well as one ASV from *Bacteroidia* and one from *Polyangia*, in the rhizosphere bacterial community. Several studies have documented that *Citrobacter*—widely detected in WW and treated effluents (Shin et al. 2022) and *Rheinheimera* species (both *Enterobacterales*) harbour multiple ARGs, often carried on conjugative plasmids, making them notable reservoirs and potential vectors of antimicrobial resistance in WW-irrigated contexts (Belachew et al. 2018, Tesfaye et al. 2019, Fu et al. 2020a, 2020b, Ota et al. 2022, Hem et al. 2024).

The most notable shifts in the rhizosphere bacterial communities following irrigation with UWW compared to TWW were the higher relative abundances of six ASVs affiliated with *Enterobacterales, Burkholderiales, Xanthomonadales, Flavobacteriales*, and *Rhizobiales*. Notably, ASV857, assigned to the genus *Acidovorax* within *Burkholderiales*, was significantly more abundant in the rhizosphere after irrigation with UWW.

Several other ASVs showed significantly higher relative abundances in the rhizosphere following irrigation with TWW than following UWW irrigation, including ASVs from the orders *Enterobacterales, Pseudomonadales, Caulobacterales, Rhizobiales, Chloroflexales*, and *Pseudonocardiales*. This aligns with previous research from Zolti et al. (2019) demonstrating that irrigation water quality significantly shapes the rhizosphere bacterial community, with TWW irrigation promoting an *Actinomycetia* decline and a *Gammaproteobacteria* enrichment.

In cilantro phyllosphere, all analyzed ARGs and MGEs were detected, except pSK1- and pI258-like plasmids, which were either rare or below the detection limit. In contrast, Bhattacharjee et al. (2024) did not detect any ARG in MAGs from the phyllosphere of spinach and radish irrigated with municipal WW containing antibiotics using a metagenomic approach. This highlights the importance of using qPCR or complementary cultivation-based approaches to detect ARGs from low-abundant bacteria or plasmid-associated ARGs that may not be detected by metagenome analyses.

Overall, spiking, WW type, and soil type did not significantly impact the ARG and MGE profiles in the phyllosphere of cilantro. This aligns with findings by Cerqueira et al. (2019c), who reported minimal influence of irrigation water quality on ARG abundance in plants. Heyde et al. (under revision) found no statistically significant reduction of trimethoprim and sulfamethoxazole concentrations in cilantro shoots irrigated with TWW instead of UWW. In our study, spiking the irrigation water led to increased relative abundances of the resistance genes *ermA* and *qnrA*. Similarly, Shen et al. (2019) observed that ARG profiles were responsive to pharmaceutical exposure. The detection of ARGs on edible plants raises potential public health concerns when consumed raw by humans (Blau et al. 2018, Reid et al. 2020, Zhang et al. 2022).

The dominant phyla of the cilantro phyllosphere were *Pseudomonadota* and *Bacillota*, followed by *Actinomycetota* and *Bacteroidota*, in agreement with Sohrabi et al. 2023 Among the dominant genera were *Exiguobacterium, Pseudomonas, Pantoea*, and *Acinetobacter*—bacteria known for producing antimicrobial substances promoting plant health and enhancing nutrient cycling (Raio and Puopolo 2021, Lv et al. 2022, Marfetán et al. 2023, Mujumdar et al. 2023, Alattas et al. 2024). However, some species within these genera are known as important plant pathogens (Coutinho and Venter 2009, Xin et al. 2018) and human opportunistic pathogens (Antunes et al. 2014, Dutkiewicz et al. 2016, Chen et al. 2017, Sanz-García et al. 2021), posing potential risks for plant disease and human opportunistic infections (Ma et al. 2025).

In the phyllosphere, no ASVs showed a significant response to the irrigation with spiked WW or water type. However, one ASV affiliated with the genus *Massilia*, responded significantly to the soil type, showing higher relative abundance in the phyllosphere of cilantro grown in Vertisol soils compared to Leptosol soils. *Massilia* was also detected in the study of Bhattacharjee et al. (2024). They found a carbapenem resistance gene in a MAG belonging to the genus *Massilia*. It was assembled from metagenomes of bulk soil planted with spinach and irrigated with treated municipal WW.

Different 3GCR enterobacteria were cultivated from stained soil and cilantro rhizosphere of both soil types. The frequently detected *Citrobacter* strains (Ci-1a_Bx-C2) carrying *bla*_CTX-M_ and *bla*_TEM_ genes were only isolated from stained soil of both soil types and rhizosphere irrigated with TWW, either spiked or unspiked. Although 3GCR *Citrobacter* typically carry inducible chromosomally encoded AmpC beta-lactamases (*bla*_CMY_ genes) (Barlow and Hall 2002, Chenhaka et al. 2023, Duduveche 2026), *bla*_CTX-M_ and *bla*_TEM_ genes have also been reported for clinical and environmental *C. freundii* strains (Said et al. 2015, Ncir et al. 2024, Duduveche 2026, Fonton et al. 2026). Whether these ESBL genes are chromosomally or plasmid-encoded, and whether they can be transferred to other *Enterobacteriaceae*, requires further investigation (Gekenidis et al. 2020, Ncir et al. 2024).

In contrast to the DNA-based methods, which failed to detect any effects of irrigation water quality on soil bacterial communities, selective enrichment revealed that the frequently isolated *Citrobacter* Bx-2 strains—whose corresponding ASVs were detected in TWW but not in UWW—had likely been introduced into the stained soil and the rhizosphere through irrigation with TWW and persisted there. Thus, we propose an irrigation water quality effect that remained below the limit of detection of the DNA-based methods (Davidovich et al. 2025). The dominant *Citrobacter* genotypes were phylogenetically placed into the *C. freundii* complex. As pointed out by Duduveche (2026) and Wirth et al. (2026), there is an urgent need to investigate the persistence of resistant potential human pathogens, such as bacteria of the *C. freundii* complex, in the context of WW irrigation rather than focusing solely on the faecal indicator *E. coli*.

In the present study, other 3GCR enterobacteria identified as *Enterobacter, Raoultella*, and *Klebsiella*, all carrying *bla*_CTX-M_ and *bla*_TEM_ genes, were only detected in the rhizosphere, in line with their low relative abundance in the irrigation water microbiome. ESBL-producing strains of these genera have also been isolated from WW and irrigation water by Chenhaka et al. (2023), Gómez-Sanz et al. (2023), Ncir et al. (2024), Puljko et al. (2024), Ragueh et al. (2025), and Sghaier et al. (2019), suggesting WW as their source.

## Conclusion

A multi-phasic approach was employed using monolithic soil columns cultivated with cilantro to assess how antibiotics and disinfectants in WW affect the abundance and distribution of ARGs, MGEs, ARB, and bacterial communities in preferential flow path soil and plant-associated microbiomes. We demonstrate that spiking the irrigation water with antibiotics and disinfectants significantly increased the relative abundances of several ARGs and MGEs across both soil types, as well as in the cilantro rhizosphere and phyllosphere. Spiking emerged as the most important driver shaping ARG and MGE distribution in soils and preferential flow path soil. This indicates that micropollutants in irrigation water play a critical role in determining the antibiotic resistance profile and dynamics along the soil–plant continuum. Contrary to our hypothesis, irrigation with either TWW or UWW did not significantly alter the relative abundance of ARGs or MGEs in the soil, cilantro rhizosphere, or phyllosphere. While the soil type was the primary factor influencing the bacterial communities of both stained soil and cilantro rhizosphere, spiking the irrigation water also had a significant effect. However, water type alone influenced the rhizosphere bacterial community. Irrigation with different water qualities led to significant changes in specific members of the classes *Alphaproteobacteria* and *Gammaproteobacteria*. Irrigation with TWW likely introduced 3GCR *Citrobacter* Ci-1a_Bx-C2 genotype strains into the stained soil of both soil types and the rhizosphere. These findings from controlled greenhouse conditions over a short time offer a good scientific basis for large-scale field experiments under real-world agricultural scenarios. The results emphasize the importance of managing WW quality and implementing advanced treatment technologies to reduce the spread of ARGs in agricultural crops. Moreover, we demonstrated that a combined molecular and cultivation-dependent approach is required for an in-depth understanding of the effects of different irrigation water qualities on ARGs, MGEs, and bacterial communities. This is important because potential human pathogens with acquired resistances like 3GCR enterobacteria are often below the limit of detection of DNA-based bacterial community studies. Future research should involve more intensive cultivation-based characterization of ARB, as well as plasmid capture experiments in soil-plant systems. In combination with assessments of exposure to potentially selecting chemical agents, this will help elucidate the role of WW pollutants in the dissemination of environmental antibiotic resistance, including potential transmission through the food chain and associated human health risks.

## Supplementary Material

fiag087_Supplemental_Files

## Data Availability

Sequencing data (16S rRNA gene amplicon) were deposited at the Sequence Read Archive (https://www.ncbi.nlm.nih.gov/sra) under the BioProject accession PRJNA1191203 (SUB15391150). Sequencing data (Sanger-sequenced 16S rRNA genes of strains) generated in this study were deposited in GenBank under accession numbers PV492330 to PV492401.
