# Supplementary material for: Soil-plant microbiomes shaped by soil type and wastewater quality as shown by a combination of cultivation-dependent and independent techniques
Source: FEMS Microbiol Ecol. 2026 Aug 12;102(8):fiag087. doi: 10.1093/femsec/fiag087 (PMC13471283; doi:10.1093/femsec/fiag087)
Supplement: Supplementary file 1 [file fiag087_supplemental_files.zip › CE_Suppl_Figures_Tables_rev7_SGB.docx]

**Supplementary file**

**Soil-plant microbiomes shaped by soil type and wastewater quality as shown by a combination of cultivation-dependent and independent techniques**

Sara Gallego^1^, Leila Soufi^2^, Ioannis D. Kampouris^1^, Rehana Abdulvakkeil^3^, Dipen Pulami^3^, Kathia Lüneberg^4^, Benjamin J. Heyde^3,5^, Doreen Babin^1^, Christina Siebe^4^, Jan Siemens^3^, Stefanie P. Glaeser^3^, Kornelia Smalla^1^ and Elisabeth Grohmann^2^

^1^Julius Kühn Institute (JKI) - Federal Research Centre for Cultivated Plants, Institute for Epidemiology and Pathogen Diagnostics, 38104 Braunschweig, Germany

^2^Berlin University of Applied Sciences, Faculty of Life Sciences and Technology, Department of Microbiology, 13353 Berlin, Germany

^3^Justus Liebig University Giessen, iFZ Research Center for BioSystems, Land Use and Nutrition, Institute of Soil Science and Soil Conservation, 35392 Giessen, Germany.

^4^Instituto de Geología, Universidad Nacional Autónoma de México, Ciudad Universitaria, 04510 México City, México.

^5^Ruhr University Bochum, Institute of Geography, Unit Soil Sciences and Soil Resources, 44801 Bochum, Germany

*Corresponding authors: **Sara Gallego** ([**sara.gallego@julius-kuehn.de**](mailto:sara.gallego@julius-kuehn.de)) and **Elisabeth Grohmann (e-mail:** [**egrohmann@bht-berlin.de**](mailto:egrohmann@bht-berlin.de)**)**

**Table S1**. Irrigation volumes applied throughout the experiment and soil water volumes at the end of the experiment for Vertisol and Leptosol columns.

|  | Vertisol | Leptosol |
| --- | --- | --- |
| ***Irrigation volume (Liter/m^2^)*** |  |  |
| Mean | 474 | 357 |
| Median | 475 | 357 |
| Stddev | 7 | 0 |
| ***Soil water volume 0-20 cm (Liter/m^2^)*** |  |  |
| Mean | 114 | 89 |
| Median | 116 | 88 |
| Stddev | 14 | 17 |
| ***Irrigation volume/soil water volume (-)*** |  |  |
| Mean | 4.2 | 4.1 |
| Median | 4.1 | 4.0 |
| Stddev | 0.6 | 0.8 |

Stddev = Standard deviation

**Table S2.** qPCR primer and probe sets used in this study.

| Target gene/plasmid group | Primers/Probes | Sequence [5´-3] | LOQ/  LOD | Annealing temp | Reference |
| --- | --- | --- | --- | --- | --- |
| Bacterial 16S rRNA gene | Bact1369F  Prok1492R  TM1389F_P | CGGTGAATACGTTCYCGG  GGWTACCTTGTTACGACTT  CTTGTACACACCGCCCGTC | 1.18·10^6^-1.98·10^6^ | 56°C | (Suzuki *et al*. 2000) |
| *ermA* | ermA-F  ermA-R  ermA-P | TCTTATCGTTGAGAAGGGAT  CTACACTTGGCTTAGGATGA  TGCAAAATCTGCAACGAGCTTTGGG | 1.35 ·10^3^  -  2.26 ·10^3^ | 55°C | (Werner *et al.* 2022) |
| *ermB* | ermBF  ermBR  ermBP | GGATTCTACAAGCGTACCTTGGA  GCTGGCAGCTTAAGCAATTGCT  CACTAGGGTTGCTCTTGCACACTCAAGTC | 4.05·10^3^-6.75·10^3^ | 60°C | (Böckelmann *et al.* 2009) |
| *inc18* | Inc18-F  Inc18-R  Inc18-P | AACTATCAAGGGGCTAATAGGG  CCACCCTTGACGGACAAATA  TTGCAAAGCTTGGGTATCAA | 6.95 ·10^4^  -  1.16 ·10^5^ | 58°C | (Soufi *et al.* 2025) |
| *intI1* | intI1-LC1  intI1-LC5  intI1-P | GCCTTGATGTTACCCGAGAG  GATCGGTCGAATGCGTGT  ATTCCTGGCCGTGGTTCTGGGTTTT | 1.19·10^5^-1.98·10^5^ | 60°C | (Barraud *et al.* 2010) |
| *korB* (IncP-1) | korB-F  korB-Fz  korB-R  korB-Rge  korB-Rd  tp_korBgz  tp_korB | TCATCGACAACGACTACAACG  TCGTGGATAACGACTACAACG  TTCTTCTTGCCCTTCGCCAG  TTYTTCYTGCCCTTGGCCAG  TTCTTGACTCCCTTCGCCAG  TSAGGTCGTTGCGTTGCAGGTTYTCAAT  TCAGYTCRTTGCGYTGCAGGTTCTCGAT | 4.93·10^2^-8.22·10^2^ | 54°C | (Jechalke et al., 2013a, 2013b) |
| *qnrA* | qnrA-F  qnrA-F | ATTTCTCACGCCAGGATTTG  GCAGATCGGCATAGCTGAAG | 2.76·10^4^-4.60·10^4^ | 62°C | (Marti and Balcázar 2013) |
| *qnrS* | qnrS F  qnrS R | GACGTGCTAACTTGCGTGAT  TGGCATTGTTGGAAACTTG | 2.17·10^3^-3.61·10^3^ | 62°C | (Marti and Balcázar 2013) |
| *repA_N* | RepA_N-F  RepA_N-R  RepA_N-P | TCTACACAGTTGCTGAATTAATG  GGTTTTCCGAGATATAGCAG  CTGCTAAATTGCAGAGAAGG | 1.92 ·10^2^  -  3.21 ·10^2^ | 55°C | (Soufi *et al.* 2025) |
| *rep*_pI258_ | pI258-F  pI258-R  pI258-P | GTGCGAGTACTTAGGTTATGGAGA  TTCCTTGGTAAGACTTGCTTCA  TAACTCAAGAGCGTGTTGGC | 6.76 ·10^4^  -  1.13 ·10^5^ | 55°C | (Soufi *et al.* 2025) |
| *rep*_pSK1_ | pSK1-F  pSK1-R  pSK1-P | CAGGCGCTTGAAGAATTACC  CACTTACAAGTTCAAATTCGACG  CTTATCAAGGGAAAAGTCA | 6.95 ·10^1^  -  1.16 ·10^2^ | 56°C | (Soufi *et al.* 2025) |
| *sul1* | q-sul_1 653f  q-sul_1 719r  tp_sul1 | CCGTTGGCCTTCCTGTAAAG  TTGCCGATCGCGTGAAGT  CAGCGAGCCTTGCGGCGG | 1.19·10^3^-1.98·10^3^ | 60°C | (Heuer and Smalla 2007) |
| *sul2* | q_sul2 595f  q_sul2 654f  tp_sul2 614 | CGGCTGCGCTTCGATT  CGCGCGCAGAAAGGATT  CGGTGCTTCTGTCTGTTTCGCGC | 1.18·10^4^-1.96·10^4^ | 60°C | (Heuer and Smalla 2007) |
| *tetA* | tetA_qfw  tetA_qrv  tetA_tp | CCGCGCTTTGGGTCATT  TGGTCGCGTCCCAGTGA  TCGGCGAGGATCG | 1.19·10^4^-1.98·10^4^ | 60°C | (Guarddon *et al.* 2011) |
| *tetM* | tetM_qfw  tetM_qrv  tetM_tp | GGTTTCTCTTGGATACTTAAATCAATCR  CCAACCATAYAATCCTTGTTCRC  ATGCAGTTATGGARGGGATACGCTATGGY | 1.90·10^2^-3.17·10^2^ | 60°C | (Peak *et al.* 2007) |

LOQ = Limit of quantification (copies/g dry soil)

LOD = Limit of detection (copies/g dry soil)

**Table S3.** Primers and primer concentrations used for characterization of cultivated bacterial strains.

| **Target genes** | **Primers** | **Sequence (5´- … - 3´)** | **Concen-trations in PCRs [µM]** | **Primer system specific cycling conditions^a^** | **Fragment size** | **Reference** |
| --- | --- | --- | --- | --- | --- | --- |
| *gadAB* | gadA_F  gadA_R | GATGAAATGGCGTTGGCGCAAG  GGCGGAAGTCCCAGACGATATCC | 0.4  0.4 | 95°C, 30 s, 65°C, 30 s, 72°C, 1 min 30 s (30 x) | 373 bp | (Doumith et al., 2012) |
| *uidA* | uidA_F  uidA_R | CCAAAAGCCAGACAGAGT  GCACAGCACATCCCCAAAGAG | 0.3  0.3 | 95°C, 30 s, 58°C, 30 s. 72°C, 1 min 30 s (30 x) | 623 bp | (McDaniels et al., 1996) |
| BOX elements | BOXA1R | CTACGGCAAGGCGACGCTGACG | 1.0 | 94°C, 30 s, 53°C, 1 min, 70°C, 8 min (30 x) | 150 to > 3,000 bp | (Versalovic et al., 1994) |
| *bla*_CTX-M_ | CTX-M-U1  CTX-M-U2 | ATGTGCAGYACCAGTAARGTKATGGC  TGGGTRAARTARGTSACCAGAAYCAGCGG | 0.4  0.4 |  | 593 bp | (Monstein et al., 2007) |
| *bla*_TEM_ | TEM-164.SE  TEM-165.AS | TCGCCGCATACACTATTCTCAGAATGA  ACGCTCACCGGCTCCAGATTTAT | 0.4  0.4 | 95°C, 30 s, 60°C, 30 s, 72°C, 2 min (30 x) | 445 bp | (Monstein et al., 2007) |
| *bla*_SHV_ | bla-SHV.SE  bla-SHV.AS | ATGCGTTATATTCGCCTGTG  TGCTTTGTTATTCGGGCCAA | 0.4  0.4 |  | 747 bp | (Monstein et al., 2007) |
| 16S rRNA gene | EUB9F  EUB1492r | GAGTTTGATCMTGGCTCAG  CGGTTACCTTGTTACGACTT | 0.2  0.2 | 95°C, 30 s, 54°C, 30 s, 72°C, 1 min 30 s (34 x) | ~1470 bp | (Lane, 1991) |

^a^ PCRs start with an initial denaturation at 95°C for 3 min and end with a final extension at 72°C for 10 min.

Y = C/T; R = A/G; S = G/C

**Table S4.** Linear regression with bootstrap values of 16S rRNA gene copy numbers, antibiotic resistance gene and mobile genetic element relative abundances between samples from unstained soil and preferential water flow path soil (stained soil), irrigated with UWW or TWW, both unspiked and spiked with antibiotics and disinfectants. Only significant values are reported *(see attached Excel file “linear_model_regression_bootstrap_soils”)***.**

**Table S5.** Linear regression with bootstrap values of 16S rRNA gene copy numbers, ARG and MGE relative abundances in samples from unstained soil and preferential water flow path soil (stained soil), rhizosphere, and phyllosphere irrigated with UWW or TWW, both unspiked and spiked with antibiotics and disinfectants. Only significant values are reported *(see attached Excel file “linear_model _regression_bootstrap”)***.**

**Table S6.** Differentially abundant amplicon sequence variants (ASVs) in soil from preferential flow path soil, rhizosphere and phyllosphere irrigated with UWW and TWW, both unspiked and spiked with antibiotics and disinfectants (*see attached Excel file “all_diff_abundant_ASVs_all”*).

**Table S7.** Overview of cultivated and characterized 3GCR enterobacteria and *Pseudomonadaceae* strains *(see attached Excel file “Characterized bacterial 3GCR strains*”)**.**

Extended-spectrum beta-lactamase (ESBL) genes: 0: not detected; 1: detected. Phylotypes according to Figure S14; genotypes according to Fig. S15.

**Table S8.** Overview of ASVs representing genera of cultivated 3GCR enterobacteria and *Pseudomonas* spp. strains *(see attached Excel file “ASVs representing cultivated genera”*). ASV read numbers are given. ASVs marked in bold share identical 16S rRNA gene sequences with the corresponding cultivated bacterial strain(s), whereas those marked with an asterisk differ by one nucleotide**.**

| **Soil type** | **Coordinates** | **Years of UWW irrigation** | **Village** |
| --- | --- | --- | --- |
| Leptosol | 20°4'44.90"N 99°12'20.10"W | 92 | Tlaxcoapan |
| Vertisol | 20°8'30.89"N 99°10'25.26"W | 111 | Ulapa de Melchor Ocampo |

**Fig. S1.** Geographical map of the Mezquital Valley indicating the soil sampling sites, coordinates, closest villages, and years of UWW irrigation of the soils.

**Fig. S2**. Vertical section of Leptosol soil column.

**Fig. S3.** Rarefaction curves of 16S rRNA gene sequences retrieved from the sequencing analysis (n = 115).

**Fig. S4.** ARG and MGE absolute abundances (log_10_ gene copies per L) in UWW and TWW samples. Statistical significance was assessed using Wilcoxon test. The significance levels are indicated by the following asterisks: *P* < 0.05 (*), *P <* 0.01 (**), and *P <* 0.001 (***).

**Fig. S5.** Principal component analysis using ARG and MGE relative abundances from UWW and TWW. Significance of separation was assessed with PERMANOVA test (water type: R^2^ = 0.20, *P >* 0.05).

**Fig. S6.** 16S rRNA gene log_10_ copies per gram of dry soil in samples from unstained soil and preferential water flow path soil (stained soil) irrigated with UWW or TWW, both unspiked and spiked with antibiotics and disinfectants. No significant differences were observed (*P >* 0.05; linear regression with bootstrap values).

A

B

C

D

E

F

G

H

**Fig. S7.** ARG and MGE relative abundances (log_10_ copies/16S rRNA gene copies) in A) Leptosol soil samples from unstained soil, B) Vertisol soil samples from unstained soil, C) Leptosol soil samples from preferential water flow path soil (stained soil), D) Vertisol soil samples from preferential water flow path soil (stained soil), E) cilantro rhizosphere from Leptosol columns, F) cilantro rhizosphere from Vertisol columns, G) cilantro phyllosphere from Leptosol columns, H) cilantro phyllosphere from Vertisol columns irrigated with UWW or TWW, both unspiked and spiked with antibiotics and disinfectants.

**Fig. S8.** Principal Component Analysis of ARG and MGE distributions in Leptosol and Vertisol soils from unstained soil and preferential water flow path soil (stained soil) irrigated with UWW or TWW, both unspiked and spiked with antibiotics and disinfectants. Significance of separation was assessed with PERMANOVA test (microhabitat: R^2^ = 0.06, *P <* 0.05; soil type: R^2^ = 0.02, *P >* 0.05; spike level: R^2^ = 0.09, *P <* 0.05; water type: R^2^ = 0.013, *P >* 0.05).

**Fig. S9.** Multidimensional scaling analysis performed on Bray-Curtis distance matrices from 16S rRNA gene amplicon sequencing data from UWW and TWW. Significance of separation was assessed with PERMANOVA test (water type: R^2^ = 0.36, *P <* 0.05).

A

B

**Fig. S10.** Relative abundance of the five most abundant bacterial phyla (A) and classes (B) in UWW and TWW. Statistical significance was assessed using Wilcoxon test. The significance levels are indicated by the following asterisks: *P <* 0.05 (**), P <* 0.01 *(***), and *P <* 0.001 (***).

**Fig. S11.** Relative abundance of the 15 most abundant ASVs in UWW and TWW. Statistical significance was assessed using Wilcoxon test. The significance levels are indicated by the following asterisks: *P <* 0.05 (*), *P <* 0.01 (**), and *P <* 0.001 (***).

A

B

C

**Fig. S12.** Relative abundance (RA) (% reads) of the five most abundant phyla in soil from preferential water flow path soil (A), rhizosphere (B), and phyllosphere (C) irrigated with UWW and TWW, both unspiked and spiked with antibiotics and disinfectants. No significant differences were observed (*P >* 0.05; Dunn´s test with Benjamini Hochberg adjustment).

A

B

C

**Fig. S13.** Relative abundance (RA) (% reads) of the 5 most abundant genera in soil from preferential water flow path soil (A), rhizosphere (B), and phyllosphere (C) irrigated with UWW and TWW, both unspiked and spiked with antibiotics and disinfectants. No significant differences were observed (*P >* 0.05; Dunn´s test with Benjamini Hochberg adjustment).

**Fig. S14.** Phylogenetic affiliation of cultivated 3GCR enterobacteria and *Pseudomonas* spp. strains based on partial 16S rRNA gene sequences spanning approx. 830 nt of the 16S rRNA gene sequences among gene positions 104 to 934 according to the numbering of the 16S rRNA gene by Brosius et al. (1978). The phylogenetic tree was calculated in ARB (Ludwig et al., 2004) in the [All-Species Living Tree](https://pubmed.ncbi.nlm.nih.gov/21497273/) project (LTP) database (Yarza et al., 2010) using the Neighbor joining method and the Jukes Cantor evolutionary DNA model implemented in ARB. Numbers at nodes represent bootstrap values in % based on 100 replications. Only values of 70 % and above are depicted. *Enterococcus* spp. type strain sequences were used as outgroup. Bar: 0.1 base substitutions per nucleotide position. Strains cultivated in this study are given in bold. GenBank accession numbers of respective 16S rRNA gene sequences are given. Based on sequence similarities and clustering in the trees phylotypes were defined.

A

B

**Fig. S15.** Cluster analyses of cultivated bacterial strains based on genomic fingerprint patterns generated by BOX-PCR and analyzed in BioNumerics (Applied Math). BOX-PCR patterns were separated by 1.4% agarose gel electrophoresis and DNA fragments were visualized by ethidium bromide staining. Similarity matrices based on the Pearson correlation index were calculated, and cluster analysis was performed with the unweighted pair group method with arithmetic mean (UPGMA) method. A few representatives (shown in bold and highlighted in grey) were chosen from groups of strains sharing identical DNA fingerprints and subjected to 16S rRNA gene Sanger sequencing. A, 3GCR *Citrobacter*, B, other 3GCR enterobacteria.

References

Barraud O, Baclet MC, Denis F *et al.* Quantitative multiplex real-time PCR for detecting class 1, 2 and 3 integrons. *J Antimicrob Chemother* 2010;**65**(8):1642–5. https://doi.org/10.1093/JAC/DKQ167.

Böckelmann U, Dörries HH, Ayuso-Gabella MN *et al.* Quantitative PCR monitoring of antibiotic resistance genes and bacterial pathogens in three european artificial groundwater recharge systems. *Appl Environ Microbiol* 2009;**75**(1):154–63. https://doi.org/10.1128/AEM.01649-08.

Guarddon M, Miranda JM, Rodríguez JA *et al.* Real-time polymerase chain reaction for the quantitative detection of *tetA* and *tetB* bacterial tetracycline resistance genes in food. *Int J Food Microbiol* 2011;**146**(3):284–9. https://doi.org/10.1016/j.ijfoodmicro.2011.02.026.

Heuer H, Smalla K. Manure and sulfadiazine synergistically increased bacterial antibiotic resistance in soil over at least two months. *Environ Microbiol* 2007;**9**(3):657–66. https://doi.org/10.1111/J.1462-2920.2006.01185.X.

Jechalke S, Dealtry S, Smalla K *et al.* Quantification of IncP-1 plasmid prevalence in environmental: Samples. *Appl Environ Microbiol* 2013a;**79**(4):1410–3. https://doi.org/10.1128/AEM.03728-12.

Jechalke S, Kopmann C, Rosendahl I *et al.* Increased abundance and transferability of resistance genes after field application of manure from sulfadiazine-treated pigs. *Appl Environ Microbiol* 2013b;**79**(5):1704–11. https://doi.org/10.1128/AEM.03172-12.

Marti E, Balcázar JL. Real-time PCR assays for quantification of qnr genes in environmental water samples and chicken feces. *Appl Environ Microbiol* 2013;**79**(5):1743–5. https://doi.org/10.1128/AEM.03409-12.

Peak N, Knapp CW, Yang RK *et al.* Abundance of six tetracycline resistance genes in wastewater lagoons at cattle feedlots with different antibiotic use strategies. *Environ Microbiol* 2007;**9**(1):143–51. https://doi.org/10.1111/j.1462-2920.2006.01123.x.

Soufi L, Kampouris ID, Lüneberg K *et al.* Wastewater-borne pollutants influenced antibiotic resistance genes and mobile genetic elements in the soil without affecting the bacterial community composition in a changing wastewater irrigation system. *J Hazard Mater* 2025;**494**:138680. https://doi.org/10.1016/j.jhazmat.2025.138680.

Suzuki MT, Taylor LT, DeLong EF. Quantitative analysis of small-subunit rRNA genes in mixed microbial populations via 5’-nuclease assays. *Appl Environ Microbiol* 2000;**66**(11):4605–14. https://doi.org/10.1128/AEM.66.11.4605-4614.2000.

Werner KA, Schneider D, Poehlein A *et al.* Metagenomic Insights Into the Changes of Antibiotic Resistance and Pathogenicity Factor Pools Upon Thermophilic Composting of Human Excreta. *Front Microbiol* 2022;**13:**1-19. https://doi.org/10.3389/FMICB.2022.826071.

Yarza P, Ludwig W, Euzéby J *et al*. Update of the All-Species Living Tree Project based on 16S and 23S rRNA sequence analyses. *Syst Appl Microbiol* 2010;**33**(6), 291-9. https://doi.org/10.1016/j.syapm.2010.08.001
